# Overexpression of Dyrk1A Is Implicated in Several Cognitive, Electrophysiological and Neuromorphological Alterations Found in a Mouse Model of Down Syndrome

**DOI:** 10.1371/journal.pone.0106572

**Published:** 2014-09-04

**Authors:** Susana García-Cerro, Paula Martínez, Verónica Vidal, Andrea Corrales, Jesús Flórez, Rebeca Vidal, Noemí Rueda, María L. Arbonés, Carmen Martínez-Cué

**Affiliations:** 1 Department of Physiology and Pharmacology, Faculty of Medicine, University of Cantabria, Santander, Spain; 2 Institute of Biomedicine and Biotechnology (IBBITEC), (University of Cantabria- Consejo Superior de Investigaciones Científicas (CSIC) and Investigación, Desarrollo e Investigación Cantabria (IDICAN)), Santander, Spain; 3 Centro de Investigación Biomédica en Red de Salud Mental (CIBERSAM), Instituto de Salud Carlos III, Madrid, Spain; 4 Barcelona Institute of Molecular Biology, Centro Superior de Investigaciones Científicas (CSIC) and Centro de Investigación Biomédica en Red de Enfermedades Raras (CIBERER), Barcelona, Spain; The Florey Institute of Neuroscience and Mental Health, Australia

## Abstract

Down syndrome (DS) phenotypes result from the overexpression of several dosage-sensitive genes. The *DYRK1A* (dual-specificity tyrosine-(Y)-phosphorylation regulated kinase 1A) gene, which has been implicated in the behavioral and neuronal alterations that are characteristic of DS, plays a role in neuronal progenitor proliferation, neuronal differentiation and long-term potentiation (LTP) mechanisms that contribute to the cognitive deficits found in DS. The purpose of this study was to evaluate the effect of Dyrk1A overexpression on the behavioral and cognitive alterations in the Ts65Dn (TS) mouse model, which is the most commonly utilized mouse model of DS, as well as on several neuromorphological and electrophysiological properties proposed to underlie these deficits. In this study, we analyzed the phenotypic differences in the progeny obtained from crosses of TS females and heterozygous *Dyrk1A* (+/−) male mice. Our results revealed that normalization of the *Dyrk1A* copy number in TS mice improved working and reference memory based on the Morris water maze and contextual conditioning based on the fear conditioning test and rescued hippocampal LTP. Concomitant with these functional improvements, normalization of the Dyrk1A expression level in TS mice restored the proliferation and differentiation of hippocampal cells in the adult dentate gyrus (DG) and the density of GABAergic and glutamatergic synapse markers in the molecular layer of the hippocampus. However, normalization of the *Dyrk1A* gene dosage did not affect other structural (e.g., the density of mature hippocampal granule cells, the DG volume and the subgranular zone area) or behavioral (i.e., hyperactivity/attention) alterations found in the TS mouse. These results suggest that *Dyrk1A* overexpression is involved in some of the cognitive, electrophysiological and neuromorphological alterations, but not in the structural alterations found in DS, and suggest that pharmacological strategies targeting this gene may improve the treatment of DS-associated learning disabilities.

## Introduction

Down syndrome (DS) is the most common genetic cause of mental disability.

The Ts65Dn (TS) mouse is the most commonly used and best characterized segmental trisomic model of DS (see [1], [2]). This mouse exhibits several of the phenotypic characteristics present in individuals with DS, including cognitive and neuromorphological alterations and abnormal synaptic plasticity [1]–[3].

Several trisomic genes have been proposed to play a role in the cognitive impairments observed in DS individuals and in mouse models of this condition. One of these genes is dual-specificity tyrosine-(Y)-phosphorylation regulated kinase 1A (*DYRK1A*), which encodes a protein kinase that performs crucial functions in the regulation of cell proliferation and multiple signaling pathways [4], [5] that contribute to normal brain development and adult brain physiology [4], [6]. Therefore, the *DYRK1A* gene likely plays an important role in the cognitive deficits found in DS [4], [5], [7].

Evidence for the role of *DYRK1A* in various DS phenotypes is derived from studies of several segmental trisomic mouse models of DS that overexpress different sets of orthologous genes of human chromosome 21 (Hsa21), including *Dyrk1A*, and exhibit cognitive anomalies [1], [2]. Transgenic mice overexpressing DYRK1A in artificial bacterial or yeast chromosomes or carrying extra copies of the corresponding murine cDNA also exhibit altered learning and memory [8]–[11]. In addition, one of these models, YAC152F7 transgenic mice carrying 5 Hsa21 genes, including *DYRK1A,* exhibits hippocampal-dependent learning deficits [8], [12], [13]. Together, these studies suggest that trisomy of the *DYRK1A* gene may contribute to the cognitive disabilities associated with DS.

Overexpression of DYRK1A has also been implicated in the motor alterations [9], [14] found in the DS population [15]–[17]. Although most studies have failed to detect anomalies in motor function or coordination in the most commonly used segmental trisomic model of DS, i.e., the TS mouse [18]–[20], in various experimental settings, these animals exhibit hyperactivity that is indicative of altered attention [20]–[22], which can compromise their cognitive performance during multiple tasks.

The cognitive and behavioral alterations found in DS individuals and in the TS mouse model have been attributed to two primary mechanisms: hypocellularity in various brain areas such as the hippocampus, the cerebral cortex and the cerebellum, and enhanced GABA-mediated inhibition (see [1], [23]). Multiple studies suggest that overexpression of Dyrk1A might be involved in some of these phenotypic alterations. Dyrk1A overexpression inhibits cell proliferation and induces premature differentiation of neural progenitor cells in the mouse brain [24], [25]. Consistent with these results, transgenic mice carrying an extra copy of the *Dyrk1A* gene exhibited decreased neuronal density in the cerebral cortex [5]. Therefore, *Dyrk1A* overdosage is implicated in the reduction in neuronal density found in specific brain regions of individuals with DS and of TS mice.

One of the mechanisms contributing to the hypocellularity found in TS mice is impaired pre- and post-natal neurogenesis [1]. The effects of DYRK1A overexpression on the proliferation and differentiation of embryonic neural progenitors [6] suggest that the extra copy of *Dyrk1A* in the TS mouse may affect the behavior of postnatal dentate gyrus (DG) progenitor cells, thus, contributing to the alterations of hippocampal morphology and function in these mice. Increasing evidence implicates adult hippocampal neurogenesis in the establishment of long-term potentiation (LTP) and hippocampal-dependent learning and memory [26]–[28].

Another mechanism that has been proposed to underlie the cognitive function deficits of DS individuals is enhanced inhibition. Several studies have demonstrated an imbalance between GABAergic and glutamatergic synapse activity that affects LTP (see [23]). A recent study has demonstrated anomalous NMDA receptor-mediated LTP in the prefrontal cortex of mBACtgDyrk1A mice [29], resulting in excessive inhibition. Given the role of GABA and glutamate transmission in neurogenesis, LTP and cognitive function [30]–[33], an imbalance between GABAergic and glutamatergic synapse activity may also profoundly impair cognition in DS.

To evaluate the role of *Dyrk1A* in the cognitive function of TS mice and in the various mechanisms proposed to underlie these phenotypic alterations, in this study, we genetically normalized the *Dyrk1A* gene dosage in the TS mouse and demonstrated that the overexpression of this gene is involved in working and reference memory, contextual fear conditioning and hippocampal LTP and neuromorphological (cell proliferation and differentiation in the DG, as well as the density of GABAergic and glutamatergic synapse markers) abnormalities found in the hippocampus of this model of DS. In contrast, Dyrk1A overexpression did not appear to contribute to the developmental processes that lead to hippocampal structural changes (hypocellularity, DG volume or subgranular zone (SGZ) area) or in the hyperactivity, attention or motor alterations associated with DS.

## Materials and Methods

### I. Ethics Statement

The University of Cantabria Institutional Laboratory Animal Care and Use Committee approved this study (License number: UC2012/02), and the protocols were performed in accordance with the Declaration of Helsinki and the European Communities Council Directive (86/609/EEC).

### II. Experimental Animals

Mice were generated by repeatedly backcrossing B6EiC3Sn a/A-Ts (17<16>) 65Dn (TS) females with C57BL/6Ei×C3H/HeSNJ (B6EiCSn) F1 hybrid males. The Robertsonian Chromosome Resource (The Jackson Laboratory, Bar Harbor, ME, USA) provided the parental generations, and mating was performed at the animal facilities of the University of Cantabria.

TS females were crossed with *Dyrk1A*+/− heterozygous male mice bred on a mixed C57BL/6-129Ola genetic background [34] to obtain TS mice carrying a triplicated Mmu16 segment (TS +/+/+) extending from the *Mrp139* gene to the *Znf295* gene, including the *Dyrk1A* gene; mice trisomic for all of these genes but diploid for *Dyrk1A* (TS +/+/−); and euploid (CO) mice containing a normal *Dyrk1A* dosage (CO +/+).

To determine trisomy, the animals were karyotyped using real-time quantitative PCR, as previously described [35]. C3H/HeSnJ mice carry a recessive mutation that leads to retinal degeneration (Rd); therefore, all of the animals were genotyped using standard PCR to detect the Rd mutation [36]. Experiments were conducted using wt/wt or Rd1/wt animals. The *Dyrk1A*+/− mice were genotyped using PCR, as previously described [34].

Mice were housed in groups of two or three in clear plexiglass cages (20×22×20 cm) under standard laboratory conditions with a temperature of 22±2°C, a 12-hour light/dark cycle and free access to food and water. The light/dark cycle was inverted to conduct the behavioral studies during the active period of the mice.

A total of 36 male mice were evaluated for one month in the behavioral studies (12 TS +/+/+, 12 TS +/+/−, and 12 CO +/+). In each behavioral experiment, animals were evaluated one at a time. After completion of the behavioral studies, the mice were sacrificed at the age of 6–7 months; 6 animals from each group were used for the histological experiments, and the remaining animals were used for the determination of Dyrk1A protein levels. To assess LTP, we used 6 animals (5–6 months of age) from each group that had not been previously utilized in the behavioral studies. The researchers were blinded to the genotype and karyotype throughout the entire assessment. The behavioral studies were performed in the Behavioral Laboratory of the Animal Facilities in the University of Cantabria. Western blot analysis and immunohistochemical experiments were performed in the Laboratory of Neurobiology of Learning of the Department of Physiology and Pharmacology of the University of Cantabria; the electrophysiological experiments were conducted in the Laboratory of Electrophysiology located in this department of the University of Cantabria.

### III. Western blot analysis

Immunodetection of the Dyrk1A protein was performed using the hippocampi of six animals from each experimental group. Mice were euthanized by decapitation, the brains were rapidly removed, and the hippocampus and cortex were dissected. Lysates were prepared as previously described [37]. Total protein content of the samples was determined using the method of Lowry et al. [38]. Identical amounts of total protein from each sample were loaded on a 10% sodium dodecyl sulfate-polyacrylamide gel, electrophoresed and transferred to a polyvinylidene difluoride (PVDF) membrane (Bio-Rad) using a Mini Trans-Blot Electrophoretic Transfer Cell (Bio-Rad). Nonspecific binding of antibodies was prevented by incubating the membranes in TBST buffer (10 mM Tris-HCl, pH 7.6, 150 mM NaCl, and 0.05% Tween 20) containing 5% nonfat milk powder. The blots were incubated with a mouse monoclonal anti-DYRK1A antibody (1∶250; Abnova Corporation, Taipei, Taiwan, ROC) diluted in TBST containing 2% bovine serum albumin (BSA) overnight at 4°C. After extensive washing with TBST, the blots were incubated with a goat anti-mouse IRDye 800CW antibody (1∶10.000, LI-COR Biotechnology, Lincoln, Nebraska, USA) for 1 h at room temperature. The fluorescence was detected and quantified using a LI-COR ODYSSEY IR Imaging System V3.0 (LI-COR Biotechnology, Lincoln, Nebraska, USA). The integrated optical density of the bands was determined (ImageJ software, version 1.45 s; http://rsb.info.nih.gov/ij, National Institutes of Health, MD, USA) and normalized to the background values. The relative variations between the bands of the three groups of experimental mice were calculated using an identical film. Each individual sample was evaluated in at least three independent experiments. The values were within a linear range. To ensure equal loading, the blots were reprobed using a mouse monoclonal anti-GAPDH antibody (6C5) (1∶2000; Santa Cruz Biotechnology, Santa Cruz, CA, USA).

### IV. Behavioral studies

To decrease the chances of behavioral responses being altered by prior test history, the most invasive procedures were performed last. Studies were performed in the following order: motor test, rotarod test, open field test, Morris water maze and fear conditioning.

#### 1. Cognitive tests

The **Morris Water Maze test** was performed to evaluate spatial learning using a circular tank of 120 cm in diameter that was filled with water maintained at 22–24°C. Powdered milk was added to give the water an opaque appearance. Inside the tank, a platform was hidden 1 cm below the water level.

The animals were tested in 12 consecutive daily sessions consisting of 8 acquisition sessions (platform submerged) followed by 4 cued sessions (platform visible). All of the trials were videotaped using a camera located 2 m above the water level. An Anymaze computerized tracking system (Stoelting, Wood Dale, IL, USA) was used to analyze the swimming trajectories, measure the escape latency, the path efficacy (an index of the similarity between the trajectory of each animal and the shortest trajectory to the escape platform), thigmotaxis and the swimming speed per animal for each trial.

In this study, we used the platform relocation protocol. This paradigm has been consistently used to assess learning and memory abilities in various experimental models of disease [39]–[42].

In the acquisition sessions (S1–S8), the platform was hidden 1 cm below the water level. During these sessions the platform position was changed every day.

In this protocol, the within-session (between-trial) performance analysis provides a measure of working memory (i.e., the animals search for the platform during the first trials, encode its location and then remember it on the remaining trials of the day). Therefore, effective performance required the retrieval of recent information, or “working memory” [43]. However, for each new session, the animal must learn a new platform position. Therefore, a “between-session” performance analysis provides a measure of “reference memory”. As the sessions progress, the animal must construct a stable long-term memory representation of the environment (i.e., a “coordinate” map), which can be considered the reference memory component of the task, separate from the representation of the most recent location in which the hidden platform was located. Moreover, throughout the sessions, the animal must learn to inhibit maladaptive behaviors, such as swimming along the walls, and to choose a strategy to search for the platform in the tank. Therefore, “between-session” performance analysis also provides a measure of the learning and memory abilities of the animals.

Each of the 8 acquisition sessions and 4 cued sessions (one session per day) consisted of four pairs of trials at 30–45 min intervals. In each pair of trials, the mice were randomly started from one of the four positions (N, S, E or W), which were held constant for both trials. The first trial of the pair was terminated when the mouse located the platform or when 60 s had elapsed; the second trial commenced following a period of 20 s, during which the mouse was allowed to remain on the platform. Several fixed visual cues outside the maze were constantly visible from the pool.

During the 4 cued sessions, the platform was visible; the water level was 1 cm below the top of the platform, and its position was indicated with a flag. Eight trials were performed during each session according to an experimental procedure that was identical to the procedure used in the acquisition sessions.

For the **Fear conditioning** experiment the procedure was performed as described by Salehi et al. [44]. Briefly, contextual and tone-cued fear conditioning tests were performed using the Fear Conditioning apparatus (Stoelting) and the AnyMaze Video Tracking System (Stoelting). The mice underwent three days of testing: a training day, a tone-cued in a novel context testing day and a contextual testing day. On the first day, the mice went through the training session. Prior to conditioning, each mouse was allowed to explore the test chamber for 3 min (baseline activity). Then, each mouse received five tone-shock pairings. The shock (0.5 mA, 50 Hz, 2 sec) was delivered 18 sec after the end of the tone (70 dB, 2 kHz, 20 sec). Therefore, there was an empty trace interval interposed between the tone and the shock for each conditional stimulus-unconditional stimulus pairing. On the second day (the tone-cued testing day), each mouse was placed in a novel context (new olfactory and visual cues) for 3 min and was subsequently presented with three tone exposures (identical to the training day) without any shocks. On the final day of the experiment, each mouse was placed in a context similar to the training day for 5 min without any tones or shocks. The freezing behavior of the mice under each condition was quantified on both testing days.

#### 2. Motor tests

A battery of motor tests was performed following the procedure described by Martínez-Cué et al. [20]. In the visual placing reflex test, the cerebellar and vestibular functions were evaluated. In 3 consecutive trials, mice were gently lowered by the tail towards a flat surface from a height of 15****cm. The response of forepaw extension was scored on a 0–4 scale (4: animal extends the forepaws when placed at the highest height; 3: forepaws extended before touching the surface with vibrissae; 2: forepaws extended after vibrissae touched the surface; 1: forepaws extended after the nose touched the surface; and 0: no extension).

Grip strength was assessed by quantifying the resistance to separation from the aluminum bars (2 mm) of a lid when pulled by the tail (0: no resistance; 1: slight; 2: moderate; 3: active; and 4: extremely active resistance).

To evaluate the equilibrium of the mice, four 20-s trials of balance were performed on an elevated (40 cm high), horizontal (50 cm long) rod. Trials 1 and 2 were performed on a flat wooden rod (9 mm wide); trials 3 and 4 were performed on a cylindrical aluminum rod (1-cm diameter). In each trial, the animals were placed in a marked central zone (10 cm) on the elevated rod. A score of 0 was given if the animal fell within 20 s, 1 if it remained within the central zone for >20 s, 2 if it left the central zone, and 3 if it reached one of the ends of the bar.

Prehensile reflex (three 5-s trials) was measured from the ability of the animal to remain suspended by the forepaws by grasping an elevated horizontal wire (2 mm in diameter). The maximum possible score was achieved when the animal remained suspended by the forepaws in all three trials (one point per trial). Traction capacity was simultaneously scored by assessing the number of hind limbs that the animal raised to reach the wire (0: none; 1: one limb; and 2: two limbs).

Motor coordination was evaluated using a **rotarod** device (Ugo Basile, Comerio, Italy), which consists of a 37-cm-long, 3-cm diameter plastic rod that rotates at different speeds. In a single session, 5 trials with a maximum duration of 60 s each were performed. In the first four tests, the rod was rotated at constant speeds of 10, 20, 30 or 50 rpm, respectively. The last trial consisted of an acceleration cycle, in which the rod rotated progressively faster, and the animal had to adapt to the growing demands of the test. The length of time that each animal remained on the rotarod during the acceleration cycle was recorded.

Exploratory behavior and anxiety were assessed using a square-shaped **open field** (55 cm×55 cm, surrounded by a 25-cm-high fence) divided into 25 equal squares. The mice were placed in the center of the field, and the number of vertical (rearing) activities and horizontal crossings (from square to square, subdivided into center vs. peripheral crossings) were scored in a single 5-min trial.

### V. Long-term potentiation

The mice were decapitated, and the brains were rapidly removed. The hippocampi were dissected, and 400-µm slices were generated using a tissue chopper. The slices were allowed to recover for 1 hour in an interface chamber at room temperature in artificial cerebral spinal fluid (ACSF) containing 120 mM NaCl, 3.5 mM KCl, 2.5 mM CaCl_2_, 1.3 mM MgSO_4_, 1.25 mM NaH_2_PO_4_, 26 mM NaHCO_3_ and 10 mM D-glucose saturated with 95% O_2_ and 5% CO_2_. Field excitatory postsynaptic potentials (fEPSPs) were recorded from the CA1 stratum radiatum using a glass micropipette (1–4 MΩ) containing 2 M NaCl, and the Schaffer collaterals were stimulated using insulated bipolar platinum/iridium electrodes located >500 µm from the recording electrode. The stimulus strength was adjusted to evoke fEPSPs equal to 50% of the relative maximum amplitude without a superimposed population spike. After stable baseline recordings (100-µs pulse duration, 0.033 Hz), long-term potentiation (LTP) was induced with TBS (10 trains of 5 pulses at 100 Hz and intervals of 200 ms). The duration of the stimulation pulses was doubled during tetanus. fEPSPs were amplified, bandpass filtered (1 Hz-1 kHz) and stored in a computer using the Spike2 software program (Cambridge Electronic Design, Cambridge, UK). For the analysis, the fEPSP slopes were expressed as a percentage of the recorded baseline values. The results from several slices were expressed as the mean value ± SEM.

### VI. Histological and stereological procedures

The animals were deeply anesthetized with pentobarbital (50 mg/kg) and transcardially perfused with saline followed by 4% paraformaldehyde. Subsequently, the brains were postfixed in 4% paraformaldehyde overnight at 4°C and transferred to 30% sucrose. The brains were frozen in dry ice and coronally sliced in a cryostat (50-µm thick sections). Series of brain slices comprising 1 section of every 9 were used for the immunohistochemistry protocol. A 1-in-9 series of coronal sections was randomly selected and subjected to Nissl staining using the Cavalieri method, as previously described [45], to calculate the SGZ area and the DG volume.

#### 1. Granule cell counts

The cells in the hippocampal granule cell layer (GCL) were counted in sections stained with 4′6-diamidino-2-phenylindole (DAPI, Calbiochem, Billerica, MA, USA; 1∶1,000) for 10****min in phosphate buffer (PB). The cell counts were obtained using a confocal microscope coupled to a physical dissector system, as previously described [45]. Six dissector locations in each series were evaluated. In the selected locations, the confocal microscope was randomly directed toward a previously established position within the GCL. Subsequently, a series of confocal images was serially recorded according to the general principles of the physical dissector and the unbiased stereology methods. The confocal images were analyzed using the ImageJ software. The cells were counted cell-by-cell using a digital tablet upon the first appearance of labeled cells in each series of confocal images. The software generated the total number of cells when the dissector brick was completed. To count the total number of cells in the GCL, a square dissector frame was randomly situated inside the GCL. To obtain the number of cells per unit of volume (cell density), the obtained cell number was divided by the reference volume of the dissector; this parameter is the volume of a prism formed by the area of the frame multiplied by the height of the dissector.

#### 2. Cell proliferation in the SGZ of the DG (Ki67 immunofluorescence)

Ki67 immunofluorescence was performed to identify proliferating cells. The sections were preincubated in PBTBSA (PB containing 0.5% Triton X-100 and 0.1% BSA), and immunohistochemistry was performed, as previously described [45]. Briefly, free-floating sections were incubated with a primary rabbit anti-Ki67 antibody (1∶750; Abcam, Cambridge, UK) for two days at 4**°**C. Subsequently, the slices were incubated overnight at 4**°**C with a secondary antibody diluted 1∶1,000 (donkey anti-rabbit-Alexa Fluor 488; Molecular Probes, Eugene, OR, USA). The sections were counterstained with DAPI and mounted on gelatin-covered slides for analysis and imaging. The total number of Ki67-positive cells was counted under an optical fluorescence microscope (Zeiss Axioskop 2 Plus, 40x objective) using the optical dissector method, as previously described [45].

#### 3. Neuronal differentiation (DCX and CLR immunofluorescence)

One-in-nine series of 50-µm sections were used for the determination of the density of cells expressing immature markers (doublecortin (DCX) and/or calretinin (CLR)). The sections were initially preincubated in PBTBSA, and dual immunohistochemistry was subsequently performed. A goat anti-doublecortin antibody (1∶250; Santa Cruz Biotechnology, Santa Cruz, CA, USA) and a rabbit anti-calretinin antibody (1∶3000; Swant, Switzerland) were used as primary antibodies. The primary antibodies were labeled with a donkey anti-goat Alexa Fluor 594-conjugated antibody and a donkey anti-rabbit Alexa Fluor 488-conjugated antibody, respectively (1∶1000; Alexa Fluor-conjugated antibodies purchased from Invitrogen, Carlsbad, CA, USA). The sections were subsequently imaged using a confocal microscope (Leica SP5). Quantification of the DCX- and CLR-expressing cells was performed according to stereological procedures using the physical dissector method, as previously described by Llorens-Martin et al. [45]. To evaluate the cells expressing the immature markers, six random dissectors per animal were used, comprising sections of the entire hippocampus. A series of 11 confocal images was serially recorded in each physical dissector. All of the immature cells were counted using the physical dissector, which has a square area with one side that lies on the “line” of the SGZ. By dividing the number of counted immature cells by the length of the “subgranular” line, a reliable estimate of the cell density by “unit of SGZ” was obtained. The cell density of the immature neurons is presented as either DCX+ (DCX+/CLR-) or CLR+ (DCX+/CLR+ plus DCX−/CLR+).

#### 4. Density of GABAergic and glutamatergic synapse markers (GAD65/67 and VGLUT1 immunofluorescence)

One-in-nine serial 50-µm sections of mouse brains were used to determine the density of GABAergic and glutamatergic synapse markers. The sections were initially preincubated in PBTBSA, and dual immunohistochemistry was subsequently performed. The glutamatergic and GABAergic boutons were labeled using a guinea pig anti-vesicular glutamate transporter 1 antibody (VGLUT1, 1∶2,500; Millipore, Billerica, MA, USA) and a rabbit anti-glutamic acid decarboxylase 65/67 antibody (GAD65/67, 1∶350; Millipore, Billerica, MA, USA) respectively. This labeling was followed by additional labeling with Alexa Fluor 488-conjugated goat anti-guinea pig IgG (1∶1,000; Invitrogen, Carlsbad, CA, USA) and a donkey anti-rabbit Alexa Fluor 594-conjugated antibody (1∶1,000; Invitrogen, Carlsbad, CA, USA).

Measurements were performed on images obtained using a confocal microscope (Leica SP5). For each synapse marker, four sections per animal were used, constituting the entire hippocampus, and one random area in the hippocampus per section was measured. Image analysis was performed using the ImageJ software as previously described [20]. Briefly, boutons with positive immunofluorescence (either VGLUT1 or GAD65/67 because these markers do not colocalize) were separately measured, applying an identical threshold to all images. The images were converted to grayscale to improve the contrast between signal and noise. The area was measured inside of a reference circle with a standard size of 325 µm^2^. The reference space was located in the inner molecular layer of the hippocampal DG, which lines the most external layer of granule neurons in the GCL. The percentage of the reference area occupied by VGLUT1- and GAD65/67-positive boutons was calculated.

### VII. Statistical Analysis

The data obtained from the MWM test experiments were analyzed using a two-way ANOVA with repeated measures (‘session×trisomy×*Dyrk1A*’ or ‘trial×trisomy×Dyrk1A’). LTP data were analyzed using a RM MANOVA (‘time×trisomy×*Dyrk1A*’). The remaining data were analyzed using a two-way (‘trisomy’×‘*Dyrk1A*’) ANOVA. The mean values for each experimental group were compared post-hoc using Bonferroni tests. All of the analyses were performed using SPSS for Windows version 21.0.

## Results

### I. Normalization of the Dyrk1A expression level improved spatial working and reference memory and contextual fear conditioning of TS mice

**Figure 1:**
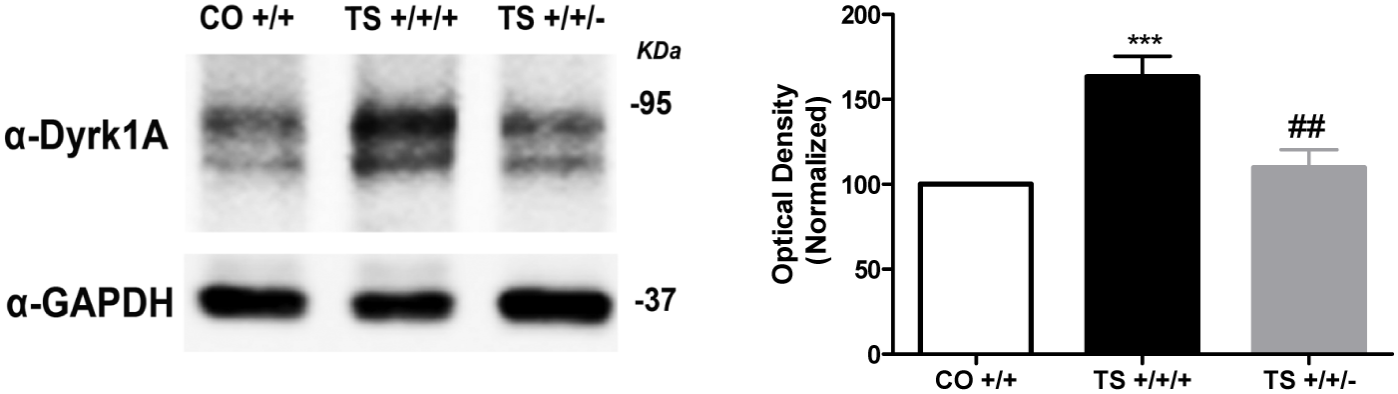
Normalization of the *Dyrk1A* copy number reduced Dyrk1A protein expression in the hippocampus of TS mice. Western blot analysis of Dyrk1A protein levels in the hippocampus of CO +/+, TS +/+/+ and TS +/+/− mice. Differences in the TS +/+/+ and TS +/+/− mice are expressed relative to the values of CO +/+ mice (defined as 100%). ANOVA ‘trisomy’: F(1,17) = 22.79, p<0.001, ‘*Dyrk1A*’: F(1,17) = 14.40, p<0.001. ***: p<0.001 TS +/+/+ vs. CO +/+; ##: p<0.01 Dyrk1A +/+/+ vs. Dyrk1A +/+/−; Bonferroni tests after significant MANOVAs.

To assess the contribution of the increased Dyrk1A expression level to various structural and functional alterations characteristic of DS, we crossed TS females and heterozygous Dyrk1A (+/−) mice and evaluated the different phenotypes in the progeny. TS +/+/+ mice exhibited a 50% increase in Dyrk1A protein expression (p<0.001) in the hippocampus. Normalization of the *Dyrk1A* gene copy number in TS +/+/− mice reduced the extent of Dyrk1A protein expression to a level similar to that found in CO +/+ mice (p<0.001; Figure **1**).

**Figure 2:**
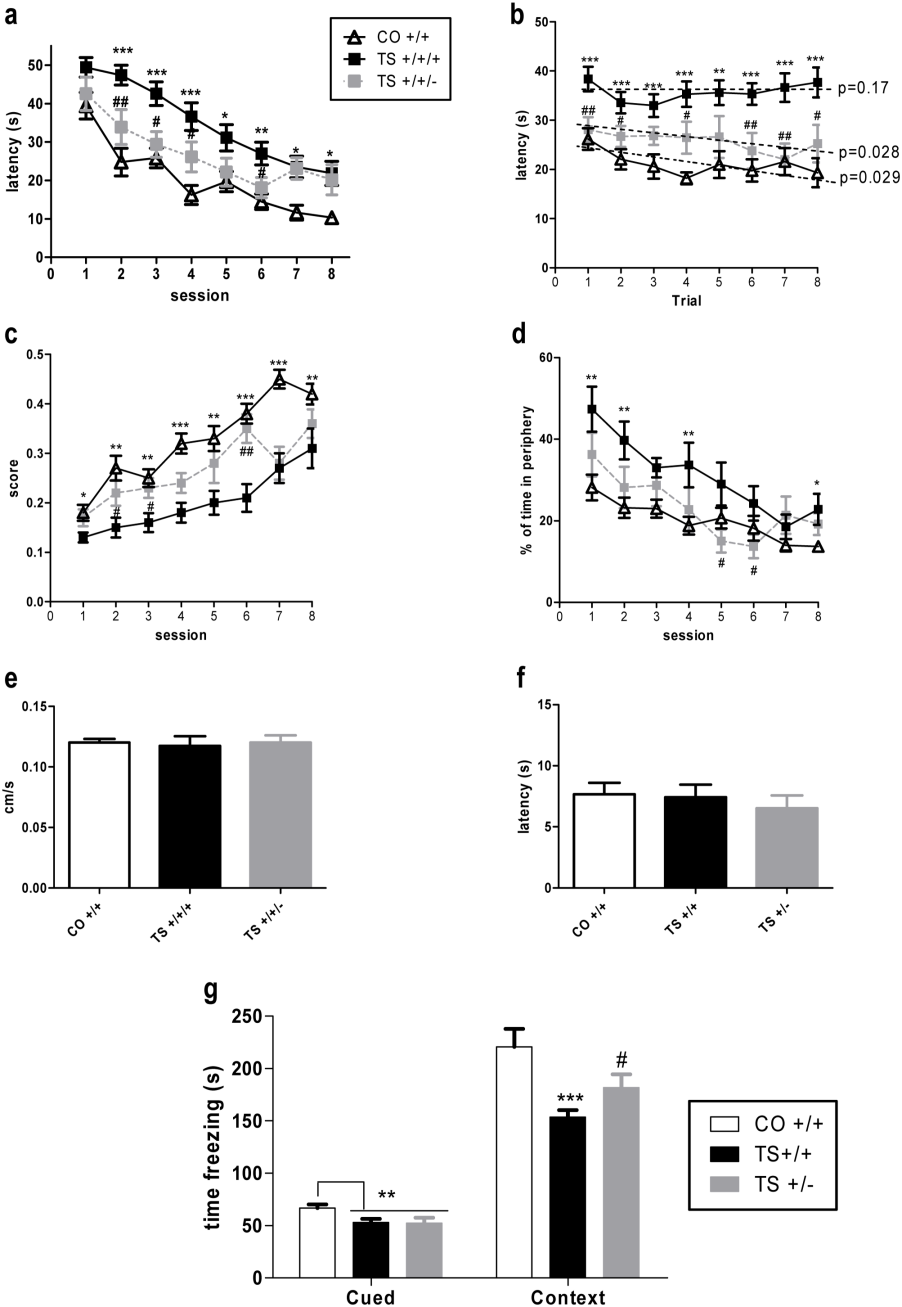
Normalization of the Dyrk1A protein expression levels in the hippocampus improved spatial working and reference memory and contextual fear conditioning in TS mice. Data are presented as the mean values ± SEM (a) of the latency to reach the platform during the acquisition sessions (between-session analysis: RM ANOVA: ‘*session*’ F(7,35) = 48.29, p<0.001; ‘*trisomy*’: F(1,35) = 44.20, p<0.001; ‘*Dyrk1A*’: F(1,35) = 7.98, p = 0.006); (**b**) of the latency to reach the platform in each trial across the 8 acquisition sessions (within-session analysis: ‘trial’ F(7,35) = 14.57, p = 0.001, p<0.001; ‘*trisomy*’: F(1,35) = 23.65, p<0.001; ‘*Dyrk1A*’: F(1,35) = 13.47, p = 0.001); (**c**) of the efficacy of the learning score (‘*session*’ F(7,35) = 22.31, p<0.001; ‘*trisomy*’: F(1,35) = 34.40, p<0.001; ‘*Dyrk1A*’: F(1,35) = 9.70, p = 0.003); (**d**) of the percentage of time spent in the periphery of the pool during the 8 sessions (‘*session*’ F(7,35) = 21.94, p<0.001; ‘*trisomy*’: F(1,35) = 13.92, p<0.001; ‘*Dyrk1A*’: F(1,35) = 8.52, p<0.001); (**e**) of the mean swimming speed (ANOVA ‘trisomy’: F(1,35) = 0.03, p = 0.84; ‘*Dyrk1A*’: F(1,35) = 0.07, p = 0.78); (**f**) of the mean latency to reach the platform in the 4 averaged cued sessions (ANOVA ‘trisomy’: F(1,35) = 0.01, p = 0.91; ‘*Dyrk1A*’: F(1,35) = 1.58, p = 0.21) in the MWM test and (**g**) of the freezing time during the cued and contextual conditioning tests (Cued conditioning: ANOVA ‘*trisomy*’: F(1,35) = 7.42, p = 0.01; ‘*Dyrk1A*’: F(1,35) = 0.01, p = 0.97; Contextual conditioning: ANOVA ‘*trisomy*’: F(1,35) = 18.64, p<0.001; ‘*Dyrk1A*’: F(1,35) = 5.54, p = 0.11). *: p<0.05, **: p<0.01, ***: p<0.001 TS +/+/+ vs. CO +/+; #: p<0.05, ##: p<0.01 Dyrk1A +/+/+ vs. Dyrk1A +/+/−; Bonferroni tests after significant MANOVAs.

After verifying that the expression level of Dyrk1A correlates with the copy number of this gene, we examined the performance of TS +/+/+, TS+/+/− and CO +/+ mice on the MWM task using a protocol that allows for assessment of both working (between-trial performance analysis) and reference (between-session performance analysis) memory. All three groups of mice were able to learn the platform position over the course of the sessions (Figure **2a**). TS +/+/+ mice exhibited an increased latency to reach the platform throughout the sessions, suggesting a profound impairment in reference memory. Remarkably, removal of one copy of *Dyrk1A* from the TS mice (TS +/+/− mice) improved their cognitive performance (p = 0.006; Figure **2a**) based on the decrease in their latency to reach the platform in sessions 1 through 6. However, during the last two sessions, the performance of the TS +/+/− mice did not differ from that of TS +/+/+ mice, indicating that the improvement in reference memory in this group of animals was only partial. Alternatively, when the performance of the different groups of mice was analyzed within each session (i.e., the latency to reach the platform in each trial across the 8 sessions), the TS +/+/+ mice exhibited a significant severe deficit in working memory compared with CO +/+ mice (p<0.001, Figure **2b**). Reducing one copy of *Dyrk1A* from the TS mice significantly improved their performance throughout the trials (p<0.001). In fact, when the slopes of the learning curves of each group of mice were analyzed separately, it was found that the TS +/+/+ mice did not learn to locate the platform position within each session because they did not reduce their latency to reach the platform over the course of the trials (p = 0.17, Figure **2b**), while both the CO +/+ (p = 0.029) and TS +/+/− mice (p = 0.028) significantly reduced their latency to reach the platform over the course of the trials; i.e., they learned the new platform position during each session. These results suggest that reduction of one copy of *Dyrk1A* ameliorated the impairment in working memory found in TS +/+/+ mice.

Next, we estimated the efficacy of learning by calculating an index of the similarity between each animal’s route to reach the platform and the shortest possible trajectory from the starting point to the platform. As illustrated in Figure **2c**, TS +/+/+ mice displayed poor efficacy in searching for the platform throughout the sessions compared to the euploid mice; however, normalization of Dyrk1A expression in TS +/+/− mice improved their efficacy in the searching for the platform throughout the 6 sessions (p = 0.003). In addition, the TS +/+/+ mice exhibited enhanced thigmotactic behavior throughout the sessions compared with the CO +/+ mice (p<0.001, Figure **2d**), and normalization of the *Dyrk1A* copy number in the TS mice significantly reduced their thigmotactic behavior based on their reduced tendency to swim in the periphery of the maze (p<0.001), indicating an improvement in their strategy of searching for the escape platform.

No significant differences in performance were detected between the three groups of mice during the cued trials (Figure **2f**) or in the swimming speed during the entire task (Figure **2e**). Therefore, the differences found during the acquisition sessions are unlikely to be due to alterations in motor function or motivation to escape from the pool.

For the fear conditioning test, the TS +/+/+ mice exhibited a deficit in remembering the association between the tone and the shock, as demonstrated by their reduced freezing time in the cued test (p = 0.01, Figure **2g**). Normalization of the *Dyrk1A* copy number did not rescue this phenotype in the TS mice. In the context conditioning test, the TS +/+/+ animals did not remember the association between the shock and the context as efficiently as the euploid mice based on their reduced freezing time during this session (p<0.001). However, this alteration was partially ameliorated in TS +/+/− animals. Although this effect was not significant compared with the euploid mice (p = 0.11; Figure **2g**), post-hoc analysis of both groups of trisomic mice indicated an enhancement of this mode of cognitive function in the TS +/+/− mice compared with the TS +/+/+ mice, as the trisomic mice with normal *Dyrk1A* gene dosage exhibited an increased freezing time compared with that of mice with three copies of this gene (p = 0.045). Therefore, normalizing the *Dyrk1A* copy number improved contextual but not cued fear conditioning in the TS mice.

### II. Normalization of the *Dyrk1A* copy number did not affect motor coordination based on the rotarod test, general activity or anxiety in the open field test or various motor functions

**Figure 3:**
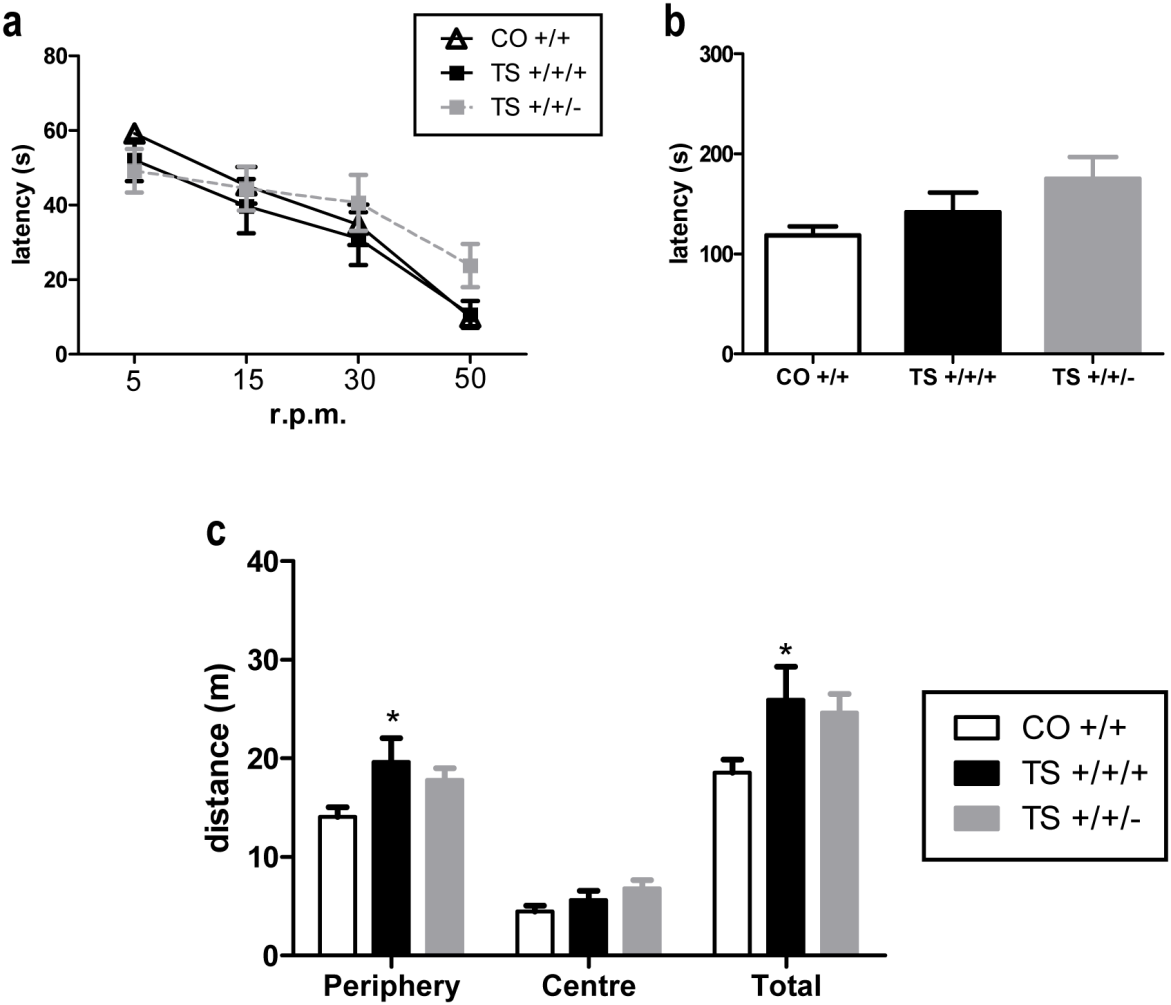
Normalization of the *Dyrk1A* copy number did not affect motor coordination in the rotarod test or the levels of general activity and anxiety in the open field test. Mean values ± SEM of the latency to fall from the rotarod at different constant speeds (**a**) and during the acceleration cycle (**b**) or of the activity performed by the three groups of mice in the open field test (**c**). Rotarod constant speeds: ANOVA ‘trisomy’: F(1,35) = 0.72, p = 0.40; ‘Dyrk1A’: F(1,35) = 1.82, p = 0.18. Acceleration cycle: ANOVA ‘trisomy’: F(1,35) = 0.72, p = 0.40; ‘Dyrk1A’: F(1,35) = 1.69, p = 0.20. Open field periphery: ANOVA ‘trisomy’: F(1,35) = 6.36, p = 0.014; ‘Dyrk1A’: F(1,35) = 0.70, p = 0.17; Center: ANOVA ‘trisomy’: F(1,31) = 2.21, p = 0.14; ‘Dyrk1A’: F(1,31) = 0.17, p = 0.68; Total: ANOVA ‘trisomy’: F(1,35) = 5.39, p = 0.023; ‘Dyrk1A’: F(1,35) = 1.77, p = 0.67. *: p<0.05 TS +/+/+ vs. CO +/+; Bonferroni tests after significant MANOVAs.

Motor function, motor coordination and general activity and anxiety were evaluated using a motor test battery, the rotarod test and the open field test respectively. Consistent with the lack of motor alterations detected based on the MWM test, the three groups of mice did not differ with respect to their motor coordination or the abilities assessed using various behavioral tests. No significant differences were found in the rotarod fall latency between the TS +/+/+, TS +/+/− and euploid mice when the rod was rotating at varying constant speeds (Figure **3a**) or at an accelerating rate (Figure **3b**). In addition, in the present study, the TS +/+/+ mice did not exhibit any alteration in equilibrium, motor coordination or most of the motor reflexes analyzed, and reducing the *Dyrk1A* copy number did not affect any of these motor functions (table **1**). These results indicate that neither trisomy nor the *Dyrk1A* copy number affected motor coordination.

**Table 1:** Motor test battery (mean scores ± SEM).

				MANOVA ‘trisomy×*Dyrk1A*’	
	CO +/+	TS +/+/+	TS +/+/−	Karyotype F(1,31)	Genotype F(2,31)
**Vision**	1.67±0.15	2.08±0.15	1.66±0.19	3.62 p = 0.066	3.29 p = 0.079
**Righting reflex**	2.91±0.08	2.91±0.08	3.00±0.00	0.01 p = 0.94	0.73 p = 0.39
**Grip strength**	1.75±0.13	1.41±0.15	1.34±0.14	2.28 p = 0.14	0.17 p = 0.68
**Latency to fall** **off a wooden bar**	40.00±0.00	39.83±0.17	40.00±0.00	1.39 p = 0.24	1.45 p = 0.23
**Latency to fall** **off an aluminum bar**	22.90±3.36	20.5±2.96	23.0±4.15	0.21 p = 0.64	0.24 p = 0.62
**Prehensile reflex**	2.41±0.35	2.42±0.28	2.67±0.19	0.010 p = 0.92	0.46 p = 0.50
**Traction capacity**	3.83±0.81	2.75±0.72	2.91±0.88	0.78 p = 0.38	0.02 p = 0.88
**Latency to fall** **off a coat hanger**	32.33±6.68	35.33±7.16	34.58±7.12	0.06 p = 0.80	0.01 p = 0.94
**Number of crossings** **on a coat hanger**	3.33±1.31	3.00±0.56	3.34±0.83	0.097 p = 0.75	0.08 p = 0.77
**Latency of arrival** **at a coat hanger**	12.50±4.65	8.58±3.18	9.42±3.62	0.53 p = 0.47	0.02 p = 0.87

To evaluate anxiety and general activity in the three groups of animals, the open field test was performed (Figure **3c**). This experimental setting has been traditionally used to analyze anxiety and general activity in mice. Although the evaluation of anxiety was not the aim of this study, numerous studies have consistently demonstrated the hyperactive behavior of TS animals exposed to this maze (see [2]). Therefore, the open field test is ideal to evaluate changes in this behavioral abnormality. On this test, no significant differences were found in anxiety based on the distance traveled by the mouse in the center of the maze. However, the TS +/+/+ mice traveled a larger distance at the periphery of the maze and a larger total distance than the CO +/+ mice, indicating hyperactivity of the trisomic animals in this test. Normalizing the *Dyrk1A* copy number slightly reduced this hyperactivity in these animals, but this effect did not reach statistical significance.

### III. Normalization of the *Dyrk1A* gene dosage rescued LTP in TS mice

**Figure 4:**
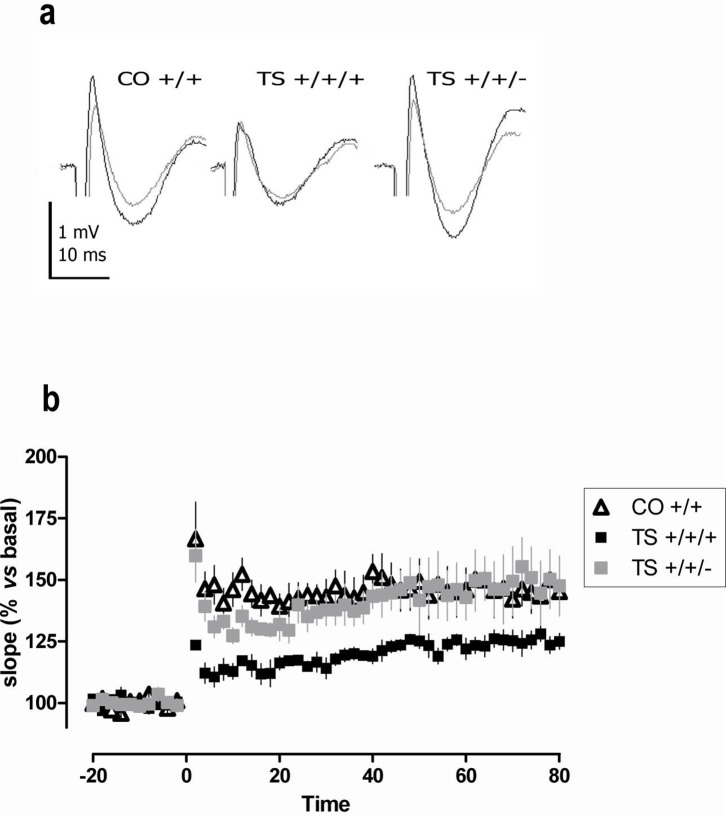
Normalization of the *Dyrk1A* copy number rescued LTP in TS mice. (**a**) Representative fEPSP traces before (gray) and after (black) TBS stimulation (top row). (**b**) Time courses of the initial slope of fEPSPs recorded from the CA1 region in hippocampal slices following stimulation of the Schaffer collateral-commissural pathway. The data are presented as the mean values ± SEM from 6 slices from 6 different mice in each group (lower row). ANOVA ‘*trisomy*’: F(1,17) = 9.34, p = 0.009; ‘*Dyrk1A*’: F(1,17) = 13.62, p = 0.003.

To evaluate the effect of the *Dyrk1A* gene dosage on the altered hippocampal plasticity of TS mice, we recorded fEPSPs in hippocampal slices obtained from the three groups of mice. A theta burst stimulus (TBS) was used to induce LTP in the CA1 region. The TS +/+/+ mice displayed a pronounced impairment of LTP (p = 0.003; Figure **4**). Importantly, normalization of the *Dyrk1A* copy number in the TS mice significantly increased the amount of LTP (TS +/+/+ vs. TS +/+/−: p = 0.008) to levels that completely abolished the deficit found in the TS +/+/+ mice (TS +/+/− vs. CO +/+: p = 0.17).

### IV. Normalization of the Dyrk1A expression level reduced the density of GABAergic and enhanced glutamatergic synapse markers in the hippocampus in TS mice

**Figure 5:**
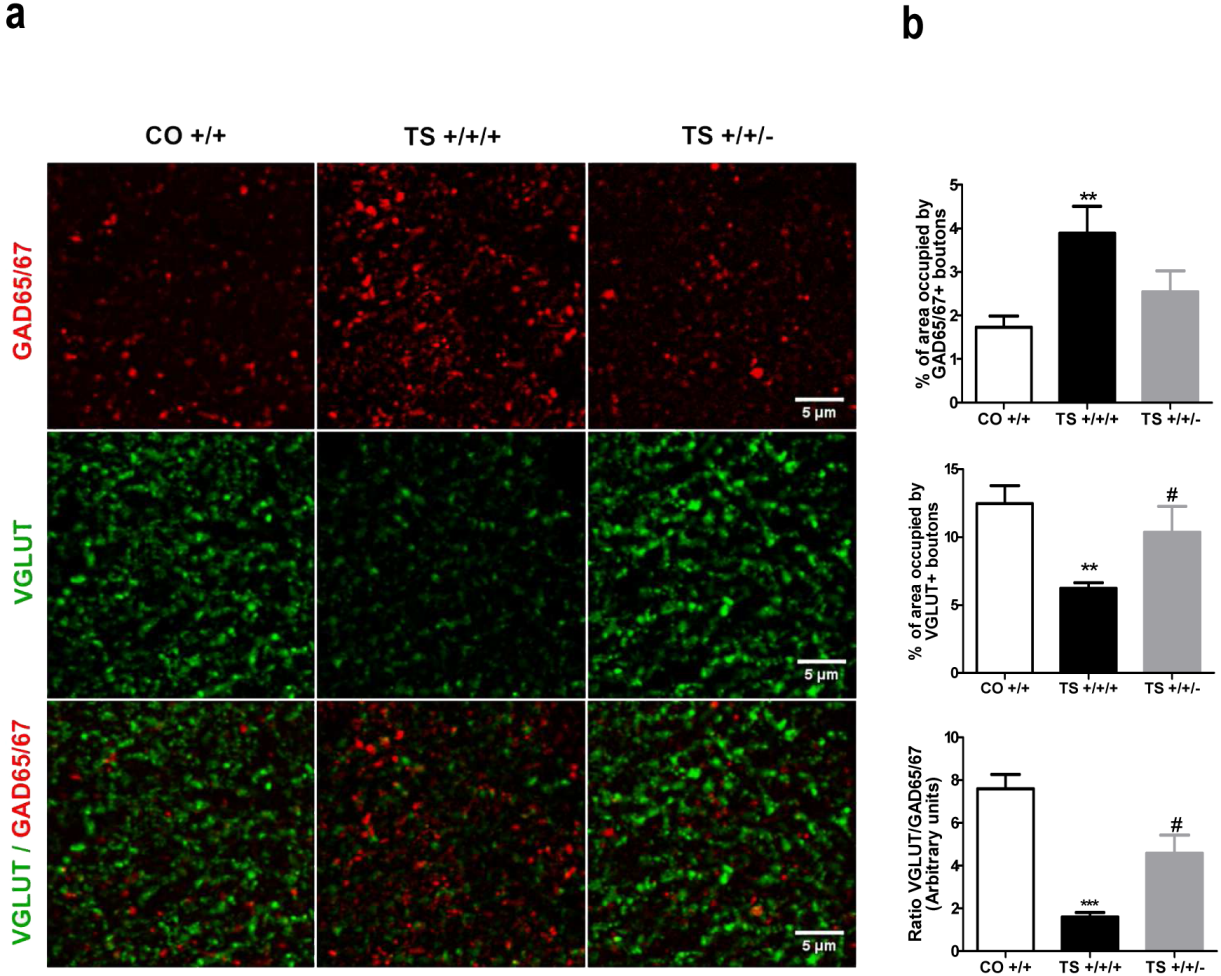
Normalization of the *Dyrk1A* copy number reduced the density of GABAergic and increased the density of glutamatergic synapse markers in the hippocampus of TS mice. (**a**) Representative images of GAD65/67, VGLUT and GAD6567/VGLUT immunostaining. (**b**) Mean values ± SEM of the percentage of area occupied by GAD65/67- (top row) and VGLUT-positive boutons (middle row) in the hippocampus of TS +/+/+, TS +/+/− and CO +/+ mice and the ratio of these areas (lower row). GAD65/67: ANOVA ‘trisomy’: F(1,17) = 10.10, p = 0.006; ‘*Dyrk1A*’: F(1,17) = 3.91, p = 0.066. VGLUT: ANOVA ‘trisomy’: F(1,17) = 10.35, p = 0.006; ‘*Dyrk1A*’: F(1,17) = 4.49, p = 0.051; Ratio GAD/VGLUT: ANOVA ‘trisomy’: F(1,17) = 37.44, p<0.001; ‘*Dyrk1A*’: F(1,23) = 9.30, p = 0.009. **: p<0.01, ***: p<0.001 TS +/+/+ vs. CO +/+; #: p<0.05 TS +/+/+ vs. TS +/+/−.

Consistent with previous studies (see [23]), in this study, the TS +/+/+ mice exhibited enhanced expression of the GABAergic synapse marker GAD65/67 and reduced expression of the glutamatergic synapse marker VGLUT in the molecular layer of the hippocampus. The TS +/+/− mice displayed a reduction in the expression of the inhibitory GABAergic marker GAD65/67 compared to the TS +/+/+ mice (Figure **5**). In addition, the decrease in the expression of the excitatory synapse marker found in the TS +/+/+ mice was ameliorated after reducing the *Dyrk1A* gene dosage, as demonstrated by the increase in the area occupied by VGLUT-positive boutons in the TS +/+/− animals (Figures **5a and b**). The calculated ratios of the expression of the two markers revealed that the TS +/+/− mice displayed a significantly improved balance of excitatory/inhibitory synapse marker expression, although these mice did not express the same levels of these markers as euploid animals (Figure **5**). These results indicate that Dyrk1A overexpression affects the density of inhibitory and excitatory synapses and suggests that it contributes to the imbalance between excitatory and inhibitory synapse marker expression found in TS +/+/+ animals. Together, these results indicate that normalizing the *Dyrk1A* copy number in TS mice improved the balance between excitatory and inhibitory synapse markers compared to trisomic mice.

### V. Normalization of the *Dyrk1A* dosage rescued neuronal proliferation and differentiation but not survival in the hippocampus of TS mice

**Figure 6:**
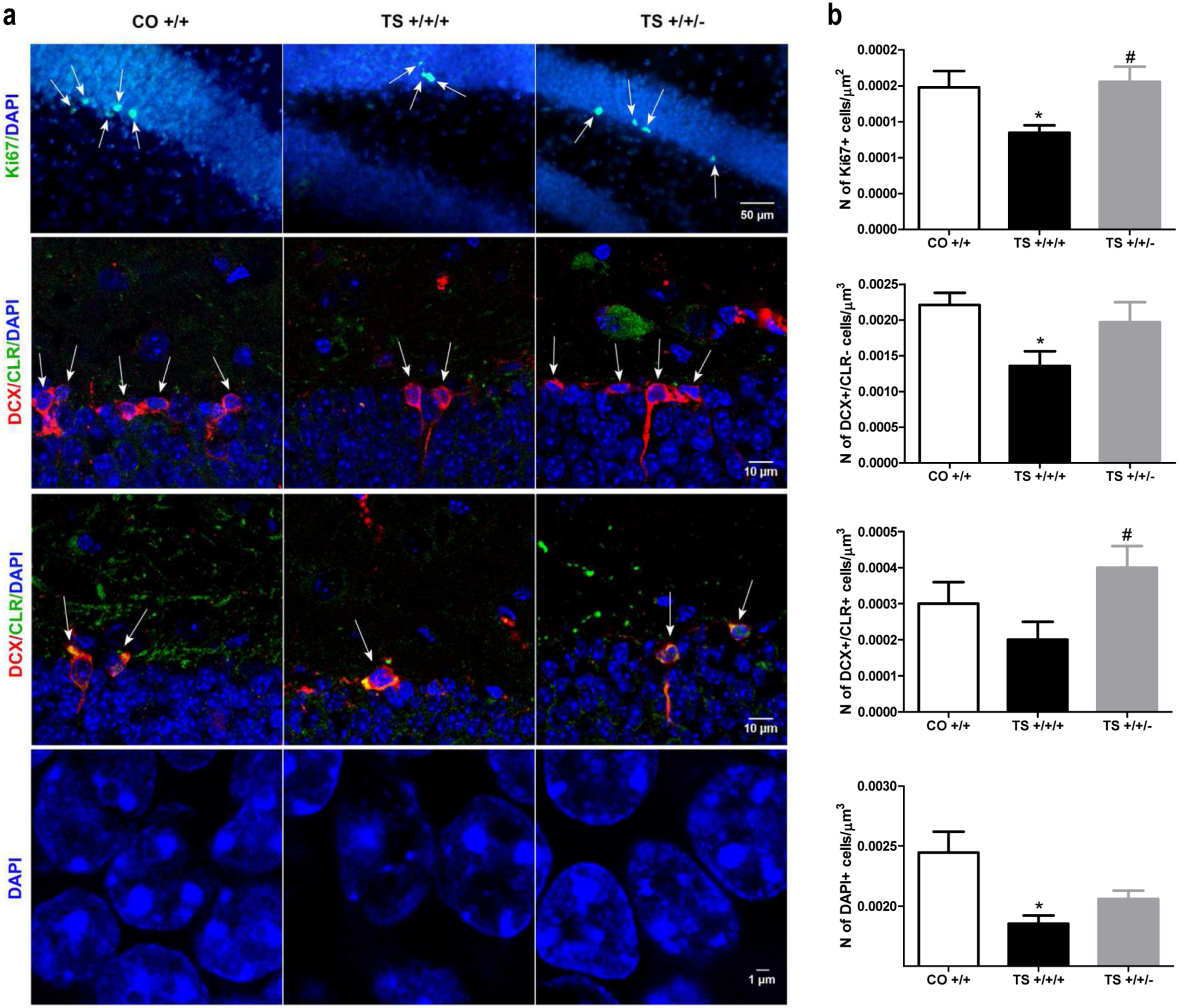
Normalization of the *Dyrk1A* gene dosage increased cell proliferation and differentiation in the DG but did not rescue the density of mature granule neurons. (**a**) Representative images of Ki67, DCX, CLR and DAPI labeling in the DG. (**b**) Mean values ± SEM of the density of Ki67-, DCX-, CLR- and DAPI-positive cells of TS mice trisomic (+/+/+) and disomic (+/+/−) for Dyrk1A and euploid (CO +/+) mice. Ki67: ANOVA ‘trisomy’: F(1,17) = 5.60, p = 0.032; ‘Dyrk1A’: F(1,17) = 7.31, p = 0.017. DCX: ANOVA ‘trisomy’: F(1,17) = 7.37, p = 0.016; ‘Dyrk1A’: F(1,17) = 3.79,; p = 0.071. CLR: ANOVA ‘trisomy’: F(1,17) = 0.94 p = 0.34; ‘Dyrk1A’: F(1,17) = 5.56, p = 0.032. DAPI: ANOVA ‘trisomy’: F(1173) = 13.43, p = 0.003; ‘Dyrk1A’: F(1,17) = 1.77, p = 0.20. *: p<0.05 TS +/+/+ vs. CO +/+; #: p<0.05 TS +/+/− vs. TS +/+/+; Bonferroni tests after significant MANOVAs.

To determine whether normalization of the *Dyrk1A* dosage would rescue the impairment in adult hippocampal neurogenesis in TS mice, we first analyzed the population of proliferating cells in the SGZ of the DG. Consistent with numerous studies [1], [20], the TS +/+/+ mice exhibited a reduced density of Ki67+ cells in the SGZ compared with the CO +/+ mice (Figures **6a and b**), indicating altered adult hippocampal cell proliferation. Remarkably, normalization of the *Dyrk1A* copy number in the TS mice completely recovered the density of Ki67+ cells.

Subsequently, we quantified neuronal differentiation using specific markers for proteins expressed by neuroblasts and immature neurons in the adult hippocampus in consecutive stages of neuronal maturation. As expected, the TS +/+/+ mice exhibited fewer DCX+ and CLR+ cells in the SGZ than the CO +/+ mice (Figures **6a and b**). However, the number of DCX+ and CLR+ cells in the TS +/+/− mice was similar to that in the control animals (Figures **6a and b**). Therefore, normalizing the expression level of Dyrk1A in TS+/+/+ animals rescued these deficits in neuronal differentiation.

When we estimated the density of mature neurons by counting the DAPI-labeled nuclei in the GCL, we found that the TS +/+/+ mice exhibited marked hypocellularity, as previously described. However, the density of the DAPI+ nuclei of the TS +/+/− mice was similar to that of the TS +/+/+ mice (Figures **6a and b** **)**. These results indicate that although normalization of the *Dyrk1A* gene dosage in TS animals improved the defects in proliferation and differentiation previously found in the hippocampus of adult TS +/+/+ mice, the newly generated neurons in TS +/+/− mice did not survive to integrate into functional circuits.

### VI. Normalization of the *Dyrk1A* gene dosage did not affect the DG volume, the SGZ area or the body weight of TS +/+/+ mice

**Figure 7:**
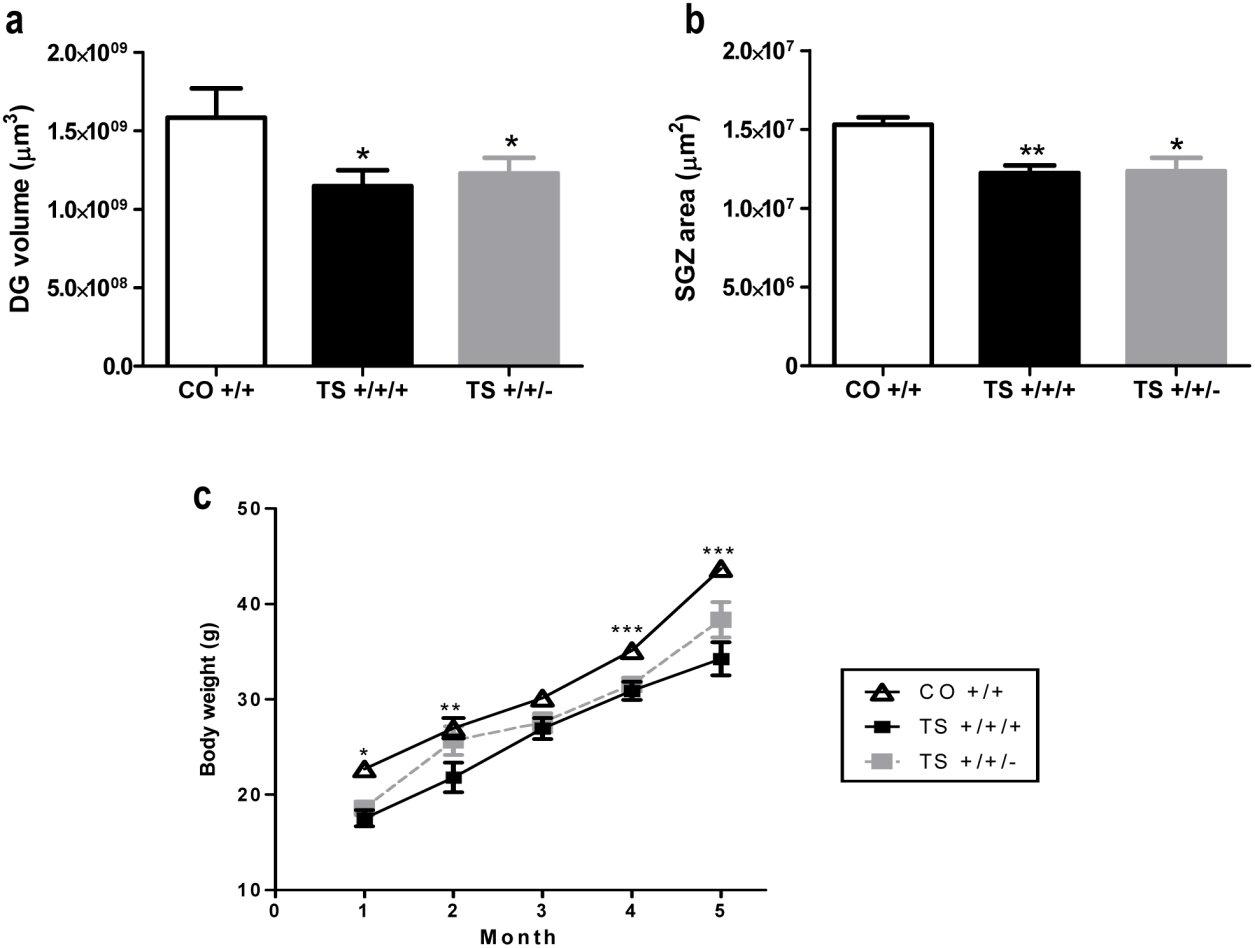
Reduction of the *Dyrk1A* gene copy number did not rescue the reduced DG volume, SGZ area or body weight in TS mice. Mean values ± SEM of the DG volume (**a**), SGZ area (**b**) and body weight (**c**) of TS +/+/+ and TS +/+/− mice and of euploid mice. DG volume: ANOVA ‘trisomy’: F(1,17) = 4.94, p = 0.043; ‘*Dyrk1A*’: F(1,17) = 0.13, p = 0.72. SGZ area: ANOVA ‘trisomy’: F(1,17) = 12.14, p = 0.003; ‘*Dyrk1A*’: F(1,17) = 0.01, p = 0.89. Body weight: ANOVA ‘trisomy’: F(1,31) = 62.98, p<0.001; ‘*Dyrk1A*’: F(1,31) = 1.31, p = 0.026. *: p<0.05, **: p<0.01, ***: p<0.001 TS vs. CO; Bonferroni tests after significant MANOVAs.

We evaluated the possible effect of *Dyrk1A* trisomy on the DG volume and the SGZ area. Adult TS +/+/+ mice exhibited a smaller DG volume and SGZ area than the CO +/+ mice (Figures **7a and b**), and these reductions were not rescued by normalization of the *Dyrk1A* gene dosage (Figures **7a and b**). This result suggests that Dyrk1A is not involved in these hippocampal morphological defects in TS +/+/+ mice.

In addition, the reduced body weight found in TS mice (from birth to the age of 5–6 months) was not significantly modified by normalization of the *Dyrk1A* gene dosage (Figure **7c**).

## Discussion

This study reveals that normalizing the *Dyrk1A* gene dosage in the TS mouse improves hippocampal-dependent learning, possibly due to the recovery of synaptic plasticity (LTP), the enhancement of adult hippocampal cell proliferation and differentiation and/or the improvement of the balance between inhibitory and excitatory synapse markers. Therefore, this study provides evidence for the role of this gene in multiple important DS phenotypes. However, normalization of the *Dyrk1A* gene dosage did not display any effect on several structural (e.g., the density of mature hippocampal granule cells, the DG volume and the SGZ area) and behavioral (hyperactivity) abnormalities found in the TS mice (see summary in Table **2**).

**Table 2:** Summary of the primary effects of the *Dyrk1A* gene dosage.

	TS +/+/+ vs. euploids	TS +/+/−
Dyrk1A protein expression in the hippocampus	↑↑ (Increased)	= (Normalized vs. euploids)
MWM test		
Reference memory	↓↓ (Impaired)	↑ (Improved vs. TS +/+/+)
Working memory	↓↓ (Impaired)	↑ (Improved vs. TS +/+/+)
Efficiency of learning	↓↓ (Impaired)	↑ (Improved vs. TS +/+/+)
Thigmotaxis	↑↑ (Increased)	↓ (Reduced vs. TS +/+/+)
Swimming speed	= (Unchanged)	= (Unchanged)
Cued learning	= (Unchanged)	= (Unchanged)
Fear conditioning test		
Cued conditioning	↓↓ (Impaired)	↓↓ (Impaired vs. euploids)
Contextual conditioning	↓↓ (Impaired)	↑ (Improved vs. TS +/+/+)
Motor function		
Motor test battery	= (Unimpaired)	= (Unimpaired)
Rotarod test	= (Unimpaired)	= (Unimpaired)
Open field test		
Hyperactivity	↑↑ (Increased)	↑ (Increased vs. euploids)
Anxiety	= (Unchanged)	= (Unchanged)
LTP	↓↓ (Impaired)	= (Rescued vs. euploids)
Synapse markers		
GAD65/67	↑↑ (Increased)	= (Rescued vs. euploids)
VGLUT	↓↓(Decreased)	= (Rescued vs. euploids)
Hippocampal histology and morphology		
Proliferation (Ki67)	↓↓ (Impaired)	= (Rescued vs. euploids)
Differentiation (DCX and CLR)	↓↓ (Impaired)	= (Rescued vs. euploids)
Survival (DAPI)	↓↓ (Impaired)	↓↓ (Impaired vs. euploids)
DG Volume	↓↓ (Reduced)	↓↓ (Reduced vs. euploids)
SGZ area	↓↓ (Reduced)	↓↓ (Reduced vs. euploids)
Body weight	↓↓ (Reduced)	↓↓ (Reduced)

Consistent with numerous reports [1], [20], TS +/+/+ mice exhibited a profound impairment in reference and working memory based on the MWM test. However, normalizing the *Dyrk1A* copy number enhanced the cognitive abilities of the TS mice. The TS +/+/− mice exhibited an improvement in the working and reference memory components of the task, in their searching strategy (i.e., the efficiency of their trajectories to reach the escape platform) and in their thigmotactic behavior compared with the TS +/+/+ mice. These effects were not due to changes in motor function, as no alteration was found in the swimming speed of TS +/+/− mice or in their performance during the cued sessions, the motor test battery or the rotarod test. These results are consistent with those of previous studies that did not find alterations in motor coordination in the rotarod test in Ts65Dn mice [18]–[20]; however, other studies have found that these mice exhibit poorer motor coordination than euploid mice in this test [46]. The discrepancies between these studies are most likely due to differences in the experimental protocol used; for example, the difficulty of the task used in the study by Costa et al. [46] was higher than that used in the other studies. In addition, conflicting results have also been found in different *Dyrk1A* transgenic mice. Although Souchet et al. [47] found a marked deficit in motor coordination in mBACtgDyrk1a mice, the TgDyrk1A model exhibited less impairment in motor learning [9], and another *Dyrk1A* model generated using a human BAC [10] does not exhibit deficits in the rotarod test. These results suggest that overexpression of Dyrk1A in a segmental trisomic mouse or in different *Dyrk1A* transgenic mice does not consistently result in motor alterations, which is most likely due to differences in the experimental protocols used. In addition, for the different *Dyrk1A* transgenic mice, the different transgenes (human or murine) and/or promoters may also account for the inconsistent results found in the rotarod test.

Finally, for Ts65Dn mice, overexpression of genes other than *Dyrk1A* in this model may affect the appearance of this phenotype. This mouse bears a partial trisomy of a segment of Mmu16, containing approximately 92 genes orthologous to Hsa21 genes [48]; in addition, the Ts65Dn mouse also carries a trisomy of ∼10 Mb of Mmu17 containing 60 genes that are not homologous to Hsa21 [49]. Thus, the set of genes that are not triplicated in DS that are trisomic in this model may also modulate or modify different phenotypes in a manner that differs from that found in single transgenic mice or other segmental trisomic mice.

Based on the fear conditioning test, the TS +/+/+ mice exhibited a deficit in both cued and contextual conditioning. Normalization of the *Dyrk1A* copy number did not rescue cued learning but partially ameliorated the alteration in contextual conditioning found in the TS +/+/+ mice. Cued conditioning primarily depends on the appropriate function of the amygdala [50]–[52], whereas the hippocampus plays a role in learning that involves context discrimination. However, the cerebellum, which is a structure that is impaired in the TS mouse (see [1]), and the entorhinal and perirhinal cortices also appear to be implicated in contextual learning [53]–[55]. Therefore, the lack of an effect of the reduction in the *Dyrk1A* gene dosage on cued learning and its limited effect in contextual learning may indicate that this gene does not affect the function of these additional brain areas.

Several studies have implicated *Dyrk1A* overexpression in the cognitive phenotype detected in animals carrying extra copies of this gene [12], [13] or in segmental trisomic mice, which also overexpress the *Dyrk1A* gene [1], [2]. A recent study [7] revealed that normalization of the Dyrk1A expression level exclusively in the hippocampus by injecting a viral vector containing inhibitory Dyrk1A shRNA (AAV2/1-shDyrk1A) did not improve the learning abilities of TS mice; however, this intervention enhanced their search strategy during the MWM test. The discrepancy between these results and the partial rescue of the performance on the MWM test found in the present study may be explained by the tissues in which Dyrk1A expression was normalized in each study. The reference and working memory components and the long-term consolidation process in the spatial learning component of the MWM test are dependent on the integrity of not only the hippocampus but also the prefrontal cortex [56]–[59], which is a structure that is also affected in DS [60]. In this study, the *Dyrk1A* gene dosage was also normalized in the cortex and, presumably, in all tissues in which Dyrk1A is overexpressed. Therefore, Dyrk1A normalization may result in more efficient learning.

Additional support for the role of *Dyrk1A* in the altered cognitive abilities found in DS mouse models comes from studies demonstrating that the pharmacological inhibition of this gene using epigallocatechin gallate (EGCG), improves hippocampal-dependent learning and thigmotaxis in TgDyrk1A and Ts65Dn mice [11], [61]. In addition, in a pilot clinical trial of individuals with DS, administration of EGCG enhanced their accuracy in visual memory recognition and spatial working memory, suggesting a positive effect of this compound on the prefrontal system [11]. Interestingly, a recent report implicated Dyrk1A overexpression in the structural and functional anomalies of the prefrontal cortex [29]. Therefore, the cognition-enhancing effects of normalization of the Dyrk1A expression level may be due to its effects on the hippocampal-prefrontal functional networks that support memory-related functions.

TS +/+/+ mice are hyperactive under conditions that typically provoke caution in euploid mice, such as the open field test, and this hyperactivity is likely due to attentional deficits [21], [22]. In the present study, normalizing the level of Dyrk1A expression did not rescue this phenotype in TS mice; this lack of rescue might contribute to the incomplete improvement in cognitive function found in TS +/+/− animals. In contrast to the present results, Ortiz-Abalia et al. [14] demonstrated that normalization of the Dyrk1A expression level in the striatum via injection of an adeno-associated virus type 2-mediated *Dyrk1A* RNA inhibitor (AAV-shDyrk1A) rescued motor deficits and attenuated hyperactivity in TgDyrk1A mice. In the present study, the lack of an effect of reducing the level of Dyrk1A expression on the hyperactivity of trisomic animals might indicate that other genes might play a role in this phenotypic alteration.

Among the putative mechanisms that mediate the cognitive-enhancing effects of normalizing Dyrk1A expression are the improvement of synaptic efficacy and the amelioration of the neuromorphological deficits found in TS mice. Altered synaptic efficacy is a predominant mechanism underlying cognitive disturbances in trisomic animals. Hippocampal LTP, which is a substrate of learning and memory, is altered in TS mice, in other mouse models of DS and in mouse models that overexpress *Dyrk1A* [1], [10], [29]. In this study, normalization of the *Dyrk1A* copy number in the TS mouse completely rescued hippocampal LTP, indicating that the extra copy of *Dyrk1A* in this mouse plays a role in the alteration in synaptic plasticity. Consistent with our results, pharmacological or genetic inhibition via administration of EGCG or injection of AAV2/1-shDyrk1A, respectively, enhances hippocampal LTP in TS mice [7], [62].

Numerous studies have demonstrated that the cognitive impairment exhibited by the TS mouse model of DS is due to an imbalance between excitatory and inhibitory neurotransmission, leading to excessive levels of neuronal inhibition (see [2], [23]). In particular, the marked reduction in LTP in the CA1 and DG areas of the hippocampus has been associated with enhanced GABA-mediated inhibition [62]–[65], as the impairments in synaptic plasticity and cognitive disturbances could be rescued in the TS mouse by administering various antagonists or negative allosteric modulators of the GABA_A_ receptor [20], [63], [64], [66], [67]. Consistent with the increased levels of GABAergic synaptic proteins in the cortex and hippocampus of TS mice [20], [65], [68], in this study, we found enhanced expression of GAD65/67, which is a marker of GABAergic synapses, and reduced expression of VGLUT, which is a marker of glutamatergic synapses. Removing one copy of the *Dyrk1A* gene reduced GAD65/67 and enhanced VGLUT expression, respectively, to levels similar to those of euploid animals. Additional support for the role of *Dyrk1A* in the enhanced inhibition comes from a recent study [47] that assessed the contribution of *Dyrk1A* gene dosage to this enhanced inhibition. This study demonstrated that increased dosage of *Dyrk1A* in mBACtgDyrk1a, Ts65Dn and Dp(16)1Yey mice carrying 3 copies of this gene led to an increased number and signal intensity of two markers of GABAergic synapses, GAD67 and VGAT. These authors propose that the increase in GAD67 staining in mBACtgDyrk1a mice could be indicative of an increase in the proportion of neurons that acquire a GABAergic phenotype during neuronal differentiation. In contrast, *Dyrk1A* (+/−) mice, which carry one functional copy of this gene, exhibited reduced expression of this inhibitory synapse marker. In addition, as observed in the present study, these authors also found increased expression of the VGLUT1 marker of excitatory synapses in the mouse models bearing three copies of *Dyrk1A* in comparison to euploid mice. These results provide additional support for the role of Dyrk1A overexpression in the overinhibition found in Ts65Dn mice.

However, in our study, the ratio of excitatory to inhibitory synapse markers was not completely restored, suggesting that additional orthologous Hsa21 genes may be involved in this imbalance. Indeed, it has been shown that triplication of the *Olig1* and *Olig2* genes, which are also in Hsa21, is also implicated in the increased number of inhibitory neurons found in the forebrains of TS mice, which is accompanied by an increase in spontaneous inhibitory postsynaptic currents in pyramidal neurons in the CA1 area [69].

TS +/+/+ mice exhibit alterations in pre- and post-natal neurogenesis (see [1]). Adult hippocampal neurogenesis plays a role in the establishment of LTP and hippocampal-dependent learning and memory [26]–[28]. Consistent with this notion, it has recently been demonstrated that restoration of neurogenesis via administration of fluoxetine, lithium or RO4938581, which is a negative allosteric modulator of the α5 subunit of the GABA_A_ receptor [3], [20], [70], to TS mice enhances their cognitive abilities. In this study, normalization of the *Dyrk1A* dosage rescued the density of proliferating (Ki67+) cells and increased the number of immature neurons (DCX+ and CLR+ cells) in TS mice. Consistent with these results, pharmacological (EGCG administration) or genetic (shRNA injection) strategies targeting Dyrk1A have been shown to abolish the proliferation and differentiation defects in iPSCs derived from an individual with DS [71]. *Dyrk1A* has been shown to interact and modulate various signaling pathways (including EGF, FGF, NGF, SHH, NFAT and Notch, among others) that play a role during different stages of cell proliferation and differentiation [6]. Therefore, normalization of the *Dyrk1A* gene dosage may aid in restoring the normal function of different molecular targets, resulting in increased adult neuronal proliferation. Because both proliferating and immature differentiating cells in the hippocampus play a role in cognitive processing during adulthood [72], [73], normalization of the Dyrk1A expression level may improve the cognitive performance of TS +/+/− animals by increasing the density of proliferating or differentiating cells. However, the *Olig1* and O*lig2* genes have also been found to modulate some of these pathways (including FGF, Notch and SHH) [74], [75]; therefore, an interplay between the effects of these genes on the different molecular targets may lead to several altered phenotypes of the Ts65Dn mouse.

In this study, the TS +/+/+ mice also displayed a reduced density of hippocampal mature granule (DAPI+) neurons. Although studies have demonstrated that DYRK1A promotes cell survival in cultures [76], in this study, normalization of the Dyrk1A expression level did not increase the density of mature surviving granule neurons in the hippocampus of TS mice. One mechanism that has been shown to enhance survival in vitro and in vivo in some tissues is the anti-apoptotic effect of DYRK1A [76]–[78]. However, the reduced cellularity found in different areas of the adult TS brain is most likely not due to changes in apoptosis [37], [79]. Thus, the anti-apoptotic effect of *Dyrk1A* might not affect the survival of mature neurons in the hippocampus when the gene dosage is normalized in TS mice. In addition, trisomy of other genes in TS mice might also be involved in the lack of a pro-survival effect after normalizing the Dyrk1A expression level.

Several studies have associated *Dyrk1A* gene dosage with brain volume [5], [10], [13]; however, consistent with the lack of an effect of normalization of the *Dyrk1A* copy number on the density of mature granule neurons, in this study, normalization of the *Dyrk1A* gene dosage did not affect the DG volume, the SGZ area or the body weight of TS animals, suggesting that other genes may be involved in these developmental alterations. Thus, the present results support the notion that *Dyrk1A* gene dosage plays a role in some of the functional, but not the structural, alterations detected in the TS mouse.

In conclusion, we demonstrate that normalization of the *Dyrk1A* copy number enhanced hippocampal-dependent learning deficits presumably by normalizing synaptic plasticity (LTP), improving the balance between excitatory and inhibitory synapse markers and/or reducing the alterations in hippocampal morphology (neural proliferation and differentiation) in TS mice. These results indicate that Dyrk1A overexpression plays an important role in several key DS phenotypes and suggest that pharmacological strategies targeting this gene or the Dyrk1A kinase could alleviate DS-associated learning disabilities.

## References

[1] Bartesaghi R, Guidi S, Ciani E (2011) Is it possible to improve neurodevelopmental abnormalities in Down syndrome? Rev Neurosci 22: 419–455. 21819263 10.1515/RNS.2011.037

[2] Rueda N, Flórez J, Martínez-Cué C (2012) Mouse models of Down syndrome as a tool to unravel the causes of mental disabilities. NeuralPlasticity 584071. doi:10.1155/2012/584071. 10.1155/2012/584071 PMC3364589 22685678

[3] Contestabile A, Greco B, Ghezzi D, Tucci V, Benfenati F, et al (2013) Lithium rescues synaptic plasticity and memory in Down syndrome mice. J Clin Invest 123: 348–361. 23202733 10.1172/JCI64650 PMC3533293

[4] Becker W, Sippl W (2011) Activation, regulation, and inhibition of DYRK1A. FEBS J 278: 246–256. 21126318 10.1111/j.1742-4658.2010.07956.x

[5] Guedj F, Lopes Pereira P, Najas S, Barallobre M-J, Chabert C, et al (2012) DYRK1A: A master regulatory protein controlling brain growth. Neurobiol Dis 46: 190–203. 22293606 10.1016/j.nbd.2012.01.007

[6] Tejedor FJ, Hämmerle B (2011) MNB/DYRK1A as a multiple regulator of neuronal development. FEBS J 278: 223–235. 21156027 10.1111/j.1742-4658.2010.07954.x

[7] Altafaj X, Martín ED, Ortiz-Abalia J, Valderrama A, Lao-Peregrín C, et al (2013) Normalization of Dyrk1A expression by AAV2/1-shDyrk1A attenuates hippocampal-dependent defects in the Ts65Dn mouse model of Down syndrome. Neurobiol Dis 52: 117–127. 23220201 10.1016/j.nbd.2012.11.017

[8] Smith DJ, Stevens ME, Sudanagunta SP, Bronson RT, Makhinson M, et al (1997) Functional screining of 2 Mb of human chromosome 21q22.2 in transgenic mice implicates minibrain in learning defects associated with Down syndrome. Nat Genet 16: 28–36. 9140392 10.1038/ng0597-28

[9] Altafaj X, Dierssen M, Baamonde C, Martí E, Visa J, et al (2001) Neurodevelopmental delay, motor abnormalities and cognitive deficits in transgenic mice overexpressing Dyrk1A (minibrain), a murine model of Down’s syndrome. Hum Mol Genet 10: 1915–1923. 11555628 10.1093/hmg/10.18.1915

[10] Ahn KJ, Jeong HK, Choi HS, Ryoo S-R, Kim YJ, et al (2006) DYRK1A BAC transgenic mice show altered synaptic plasticity with learning and memory defects. Neurobiol Dis 22: 463–472. 16455265 10.1016/j.nbd.2005.12.006

[11] De la Torre R, De Sola S, Pons M, Duchon A, de Lagran MM, et al (2014) Epigallocatechin-3-gallate, a DYRK1A inhibitor, rescues cognitive deficits in Down syndrome mouse models and humans. Mol Nutr Food Res 58: 278–288. 24039182 10.1002/mnfr.201300325

[12] Branchi I, Bichler Z, Minghetti L, Delabar JM, Malchiodi-Albedi F (2004) Transgenic mouse in vivo library of human Down syndrome critical region 1: association between DYRK1A overexpression, brain development abnormalities, and cell cycle protein alteration. J Neuropathol Exp Neurol 63: 429–440. 15198122 10.1093/jnen/63.5.429

[13] Sebrié C, Chabert C, Ledru A, Guedj F, Po C, et al (2008) Increased dosage of DYRK1A and brain volumentric alterations in a YAC model of partial trisomy 21. Anat Records 291: 254–262. 10.1002/ar.20640 18231969

[14] Ortiz-Abalia J, Sahún I, Altafaj X, Andreu N, Estivill X, et al (2008) Targeting Dyrk1A with AAVshRNA attenuates motor alterations in TgDyrk1A, a mouse model of Down syndrome. Am J Hum Genet 83: 479–488. 18940310 10.1016/j.ajhg.2008.09.010 PMC2561933

[15] McKay S M, Angulo-Barroso RM (2006) Longitudinal assessment of leg motor activity and sleep patterns in infants with and without Down syndrome. Infant Behav Dev 29: 153–168. 17138271 10.1016/j.infbeh.2005.09.004

[16] Nishizawa Y, Fujita T, Matsuoka K, Nakagawa H (2006) Contact pressure distribution features in Down syndrome infants in supine and prone positions, analyzed by photoelastic methods. Pediatr Int 48: 484–488. 16970787 10.1111/j.1442-200X.2006.02258.x

[17] Pereira K, Basso RP, Lindquist AR, da Silva AG, Tudella E (2013) Infants with Down syndrome: percentage and age for acquisition of gross motor skills. Res Dev Disabil 34: 894–901. 23291506 10.1016/j.ridd.2012.11.021

[18] Escorihuela R M, Fernández-Teruel A, Vallina IF, Baamonde C, Lumbreras MA, et al (1995) A behavioral assessment of Ts65Dn mice: a putative Down syndrome model. Neurosci Lett 199: 143–146. 8584244 10.1016/0304-3940(95)12052-6

[19] Baxter LL, Moran TH, Richtsmeier JT, Troncoso J, Reeves RH (2000) Discovery and genetic localization of Down syndrome cerebellar phenotypes using the Ts65Dn mouse. Hum Mol Genet. 9: 195–202. 10.1093/hmg/9.2.195 10607830

[20] Martínez-Cué C, Martínez P, Rueda N, Vidal R, García S, et al (2013) Reducing GABA_A_ α5 receptor-mediated inhibition rescues functional and neuromorphological deficits in a mouse model of Down syndrome. J Neurosci 33: 3953–3966. 23447605 10.1523/JNEUROSCI.1203-12.2013 PMC6619314

[21] Crnic LS, Pennington BF (2000) Down síndrome: neurophychology and animals models. Progress in Infancy Research 1: 69–111.

[22] Martínez-Cué C, Rueda N, García E, Flórez J (2006) Anxiety and panic responses to a predator in male and female Ts65Dn mice, a model for Down syndrome. Genes Brain Behav 5: 413–422. 16879635 10.1111/j.1601-183X.2005.00175.x

[23] Martínez-Cué C, Delatour B, Potier MC (2014) Treating enhanced GABAergic inhibition in Down syndrome: Use of GABA alfa5-selective inverse agonists. Neurosci Biobehav Rev, in press. Doi:10.1016/jneurobiorev.2013.12.008. 10.1016/j.neubiorev.2013.12.008 24412222

[24] Park J, Oh Y, Yoo L, Jung MS, Song WJ, et al (2010) Dyrk1A phosphorylates p53 and inhibits proliferation of embryonic neuronal cells. J Biol Chem 285: 31895–31906. 20696760 10.1074/jbc.M110.147520 PMC2951261

[25] Yabut O, Domagauer J, D’Arcangelo G (2010) Dyrk1A overexpression inhibits proliferation and induces premature neuronal differentiation of neural progenitor cells. J Neurosci 30: 4004–4014. 20237271 10.1523/JNEUROSCI.4711-09.2010 PMC3842457

[26] Shors TJ, Townsend DA, Zhao M, Kozorovitskiy Y, Gould E (2002) Neurogenesis may relate to some but not all types of hippocampal-dependent learning. Hippocampus 12: 578–84. 12440573 10.1002/hipo.10103 PMC3289536

[27] Shors TJ, Miesegaes G, Beylin A, Zhao M, Rydel T, et al (2001) Neurogenesis in the adult is involved in the formation of trace memories. Nature 410: 372–6. 11268214 10.1038/35066584

[28] Imayoshi I, Sakamoto M, Ohtsuka T, Takao K, Miyakawa T, et al (2008) Roles of continuous neurogenesis in the structural and functional integrity of the adult forebrain. Nat Neurosci 11: 1153–1161. 18758458 10.1038/nn.2185

[29] Thomazeau A, Lassalle O, Iafrati J, Souchet B, Guedj F, et al (2014) Prefrontal deficits in a murine model overexpressing the down syndrome candidate gene dyrk1a. J Neurosci 22: 1138–1147. 10.1523/JNEUROSCI.2852-13.2014 PMC3953590 24453307

[30] Tozuka Y, Fukuda S, Namba T, Seki T, Hisatsune T (2005) GABAergic excitation promotes neuronal differentiation in adult hippocampal progenitor cells. Neuron 47: 803–815. 16157276 10.1016/j.neuron.2005.08.023

[31] Ge S, Goh EL, Sailor KA, Kitabatake Y, Ming GL, et al (2006) GABA regulates synaptic integration of newly generated neurons in the adult brain. Nature 439: 589–593. 16341203 10.1038/nature04404 PMC1420640

[32] Earnheart JC, Schweizer C, Crestani F, Iwasato T, Itohara S, et al (2007) GABAergic control of adult hippocampal neurogenesis in relation to behavior indicative of trait anxiety and depression states. J Neurosci 27: 3845–3854. 17409249 10.1523/JNEUROSCI.3609-06.2007 PMC2441879

[33] Bortone D, Polleux F (2009) KCC2 expression promotes the termination of cortical interneuron migration in a voltage-sensitive calcium-dependent manner. Neuron 62: 53–71. 19376067 10.1016/j.neuron.2009.01.034 PMC3314167

[34] Fotaki V, Dierssen M, Alcántara S, Martínez S, Martí E, et al (2002) Dyrk1A haploinsufficiency affects viability and casues developmental delay and abnormal brain morphology in mice. Mol Cell Biol 22: 6636–6647. 12192061 10.1128/MCB.22.18.6636-6647.2002 PMC135639

[35] Liu DP, Schmidt C, Billings T, Davisson MT (2003) A quantitative PCR genotyping assay for the Ts65Dn mouse model of Down syndrome. Biotechniques 35: 1170–1174. 14682051 10.2144/03356st02

[36] Bowes C, Li T, Frankel WN, Danciger M, Coffin JM, et al (1993) Localization of a retroviral element within the rd gene coding for the beta subunit of cGMP phosphodiesterase. Proc Natl Acad Sci USA 90: 2955–2959. 8385352 10.1073/pnas.90.7.2955 PMC46215

[37] Rueda N, Flórez J, Martínez-Cué C (2013) Apoptosis in Down’s syndrome: lessons from studies of human and mouse models. Apoptosis 18: 121–134. 23224708 10.1007/s10495-012-0785-3

[38] Lowry OH, Rosenbrough NJ, Farr AL, Randall RJ (1951) Protein measurement with the Folin phenol reagent. J Biol Chem 193: 265–275. 14907713

[39] Steele RJ, Morris RG (1999) Delay-dependent impairment of a matching-to-place task with chronic intrahippocampal infusion of the NMDA-antagonist D-AP5. Hippocampus 9: 118–136. 10226773 10.1002/(SICI)1098-1063(1999)9:2<118::AID-HIPO4>3.0.CO;2-8

[40] Chen G, Chen KS, Knox J, Inglis J, Bernard A, et al (2000) A learning deficit related to age and beta-amyloid plaques in a mouse model of Alzheimer’s disease. Nature 408: 975–979. 11140684 10.1038/35050103

[41] Savonenko A, Xu GM, Melnikova T, Morton JL, Gonzales V, et al (2005) Episodic-like memory deficits in the APPswe/PS1dE9 mouse model of Alzheimer’s disease: relationships to beta-amyloid deposition and neurotransmitter abnormalities. Neurobiol Dis 18: 602–617. 15755686 10.1016/j.nbd.2004.10.022

[42] Saab BJ, Saab AMP, Roder JC (2011) Statistical and theoretical considerations for the platform re-location water maze. J Neurosci Methods 198: 44–52. 21419797 10.1016/j.jneumeth.2011.03.008

[43] Olton DS, Papas BC (1979) Spatial memory and hippocampal function. Neuropsychologia 17: 669–682. 522981 10.1016/0028-3932(79)90042-3

[44] Salehi A, Faizi M, Colas D, Valletta J, Laguna J, et al (2009) Restoration of norepinephrine-modulated contextual memory in a mouse model of Down syndrome. Sci Trans Med 18: 7ra17. 10.1126/scitranslmed.3000258 20368182

[45] LLorens-Martín MV, Torres-Alemán I, Trejo JL (2006) Pronounced individual variation in the response to the stimulatory action of exercise on immature hippocampal neurons. Hippocampus 16: 480–490. 16596582 10.1002/hipo.20175

[46] Costa AC, Walsh K, Davisson MT (1999) Motor dysfunction in a mouse model for Down syndrome. Physiol Behav 68: 211–220. 10627083 10.1016/s0031-9384(99)00178-x

[47] Souchet B, Guedj F, Sahún I, Duchon A, Daubigney F, et al (2014) Excitation/inhibition balance and learning are modified by Dyrk1a gene dosage. Neurobiol Dis 69: 65–75. 24801365 10.1016/j.nbd.2014.04.016

[48] Sturgeon X, Gardiner KJ (2011) Transcript catalogs of human chromosome 21 and orthologous chimpanzee and mouse regions. Mamm Genome 22: 261–271. 21400203 10.1007/s00335-011-9321-y

[49] Duchon A, Raveau M, Chevalier C, Nalesso V, Sharp A, et al (2011) Identification of the translocation breakpoints in the Ts65Dn and Ts1Cje mouse lines: relevance for modelling Down syndrome. Mamm Genome 22: 674–678. 21953411 10.1007/s00335-011-9356-0 PMC3224224

[50] Campeau S, Davis M (1995) Involvement of subcortical and cortical afferents to the lateral nucleus of the amygdala in fear conditioning measured with fear-potentiated startle in rats trained concurrently with auditory and visual conditioned stimuli: J Neurosci. 15: 2312–2327. 10.1523/JNEUROSCI.15-03-02312.1995 PMC6578111 7891169

[51] Goosens KA, Maren S (2001) Contextual and auditory fear conditioning are mediated by the lateral, basal, and central amygdaloid nuclei in rats. Learn Mem 8: 148–155. 11390634 10.1101/lm.37601 PMC311374

[52] Kholodar-Smith D B, Allen TA, Brown TH (2008) Fear conditioning to discontinuous auditory cues requires perirhinal cortical function. Behav Neurosci 122: 1178–85. 18823174 10.1037/a0012902

[53] Anagnostaras SG, Gale GD, Fanselow MS (2001) Hippocampus and contextual fear conditioning: recent controversies and advances. Hippocampus 11: 8–17. 11261775 10.1002/1098-1063(2001)11:1<8::AID-HIPO1015>3.0.CO;2-7

[54] Biedenkapp JC, Rudy JW (2009) Hippocampal and extrahippocampal systems compete for control of contextual fear. role of ventral subiculum and amygdala. Learn Mem 16: 38–45. 19117915 10.1101/lm.1099109 PMC2632852

[55] Sacchetti B, Baldi E, Lorenzini CA, Bucherelli C (2002) Cerebellar role in fear-conditioning consolidation. Proc Natl Acad Sci U S A 99 8406–8411. 10.1073/pnas.112660399 PMC123080 12034877

[56] Morris R (1984) Developments of a water-maze procedure for studying spatial learning in the rat. J Neurosci Methods 11: 47–60. 6471907 10.1016/0165-0270(84)90007-4

[57] Goldman-Rakic PS (1999) The physiological approach: functional architecture of working memory and disordered cognition in schizophrenia. Biol Psychiatry 46: 650–661. 10472417 10.1016/s0006-3223(99)00130-4

[58] Wang GW, Ci JX (2006) Disconnection of the hippocampus-prefrontal cortical circuits impairs spatial working memory performance in rats. Behav Brain Res 175: 329–336. 17045348 10.1016/j.bbr.2006.09.002

[59] Seamans JK, Floresco SB, Phillips AG (1995) Functional differences between the prelimbic and anterior cingulated regions of the rat prefrontal cortex. Behav Neurosci 109: 1063–1073. 8748957 10.1037//0735-7044.109.6.1063

[60] Goto Y, Yang CR, Otani S (2010) Functional and dysfunctional plasticity in the prefrontal cortex: roles in psychiatric disorders. Biol Psychiatry 67: 199–207. 19833323 10.1016/j.biopsych.2009.08.026

[61] Guedj F, Sebrie C, Rivals I, Ledru A, Paly A, et al (2009) Green tea polyphenols rescue of brain defects induced by overexpression of DYRK1A. PlosOne 4: 1–8. 10.1371/journal.pone.0004606 PMC2645681 19242551

[62] Xie W, Ramakrishna N, Wieraszko A, Hwang YW (2008) Promotion of neuronal plasticity by (-)-epigallocatechin-3-galate. Neurochem Res 33: 776–783. 17943438 10.1007/s11064-007-9494-7

[63] Kleschevnikov AM, Belichenko PV, Villar AJ, Epstein CJ, Malenka RC, et al (2004) Hippocampal long-term potentiation suppressed by increased inhibition in the Ts65Dn mouse, a genetic model of Down syndrome. J Neurosci 24: 8153–8160. 15371516 10.1523/JNEUROSCI.1766-04.2004 PMC6729789

[64] Fernandez F, Morishita W, Zuniga E, Nguyen J, Blank M, et al (2007) Pharmacotherapy for cognitive impairment in a mouse model of Down syndrome. Nat Neurosci 10: 411–413. 17322876 10.1038/nn1860

[65] Belichenko PV, Kleschevnikov AM, Masliah E, Wu C, Takimoto-Kimura R, et al (2009) Excitatory-inhibitory relationship in the fascia dentata in the Ts65Dn mouse model of Down syndrome. J Comp Neurol 512: 453–466. 19034952 10.1002/cne.21895 PMC8480942

[66] Braudeau J, Delatour B, Duchon A, Pereira PL, Dauphinot L, et al (2011) Specific targeting of the GABA-A receptor alpha5 subtype by a selective inverse agonist restores cognitive deficits in Down syndrome mice. J Psychopharmacol 25: 1030–1042. 21693554 10.1177/0269881111405366 PMC3160204

[67] Colas D, Chuluun B, Warrier D, Blank M, Wetmore DZ, et al (2013) Short-term treatment with the GABAA receptor antagonist pentylenetetrazole produces a sustained pro-cognitive benefit in a mouse model of Down’s syndrome. Br J Pharmacol 169: 963–973. 23489250 10.1111/bph.12169 PMC3696321

[68] Pérez-Cremades D, Hernández S, Blasco-Ibáñez JM, Crespo C, Nacher J, et al (2010) Alteration of inhibitory circuits in the somatosensory cortex of Ts65Dn mice, a model for Down’s syndrome. J Neural Transm 117: 445–455. 20157742 10.1007/s00702-010-0376-9

[69] Chakrabarti L, Best TK, Cramer NP, Carney RS, Isaac JT, et al (2010) Olig1 and Olig2 triplication causes developmental brain defects in Down syndrome. Nat Neurosci 13: 927–934. 20639873 10.1038/nn.2600 PMC3249618

[70] Bianchi P, Ciani E, Guidi S, Trazzi S, Felice D, et al (2010) Early pharmacotherapy restores neurogenesis and cognitive performance in the Ts65Dn mouse model for Down syndrome. J Neurosci 30: 8769–8779. 20592198 10.1523/JNEUROSCI.0534-10.2010 PMC6632890

[71] Hiaoui Y, Grad I, Letourneau A, Sailani MR, Dahoun S, et al (2014) Modelling and rescuing neurodevelopmental defect of Down syndrome using induced pluripotent stem cells from monozygotic twins discordant for trisomy 21. EMBO Mol Med 6: 259–277. 24375627 10.1002/emmm.201302848 PMC3927959

[72] Garthe A, Kempermann G (2013) And old test for new neurons: refining the Morris water maze to study the functional relevance of adult hippocampal neurogenesis. Front Neurosci 7: 63. 23653589 10.3389/fnins.2013.00063 PMC3642504

[73] Pérez-Domper P, Gradari S, Trejo JL (2013) The growth factors cascade and the dendrito−/synapto-genesis versus cell survival in adult hippocampal neurogenesis: the chicken or the egg. Ageing Res Rev 12: 777–785. 23777808 10.1016/j.arr.2013.06.001

[74] Schebesta M, Serluca FC (2009) Olig1 expression identifies developing oligodendrocytes in zebrafish and requires hedgehog and notch signaling. Dev Dynamics 238: 887–898. 10.1002/dvdy.21909 19253391

[75] Tiso N, Filippi A, Benato F, Negrisolo E, Modena N, et al (2009) Differential expression and regulation of Olig genes in zebrafish. J Comp Neurol 515: 378–396. 19425111 10.1002/cne.22054

[76] Guo X, Williams JG, Schug TT, Li X (2010) DYRK1A and DYRK3 promote cell survival through phosphorylation and activation of SIRT1. J Biol Chem 285: 13223–13232. 20167603 10.1074/jbc.M110.102574 PMC2857074

[77] Laguna A, Barallobre MJ, Marchena MÁ, Mateus C, Ramírez E, et al (2013) Triplication of DYRK1A causes retinal structural and functional alterations in Down syndrome. Hum Mol Genet 22: 2775–2784. 23512985 10.1093/hmg/ddt125

[78] Laguna A, Aranda S, Barallobre MJ, Barhoum R, Fernández E, et al (2008) The protein kinase DYRK1A regulates caspase-9-mediated apoptosis during retina development. Dev Cell 15: 841–853. 19081073 10.1016/j.devcel.2008.10.014

[79] Rueda N, Flórez J, Martínez-Cué C (2011) The Ts65Dn mouse model of Down syndrome shows reduced expression of the Bcl-X(L) antiapoptotic protein in the hippocampus not accompanied by changes in molecular or cellular markers of cell death. Int J Dev Neurosci 29: 711–716. 21684326 10.1016/j.ijdevneu.2011.06.001

